# Flavonoids as Modulators of the p53–Bcl-2 Axis in Cancer: Molecular Mechanisms and Therapeutic Implications

**DOI:** 10.3390/pharmaceutics18060738

**Published:** 2026-06-14

**Authors:** Julia Jankowska, Łukasz Szeleszczuk, Dariusz Maciej Pisklak

**Affiliations:** Department of Organic and Physical Chemistry, Medical University of Warsaw, Banacha 1 Street, 02-097 Warsaw, Poland; s088868@student.wum.edu.pl (J.J.); lukasz.szeleszczuk@wum.edu.pl (Ł.S.)

**Keywords:** flavonoids, apoptosis, p53, Bcl-2, cancer, cell cycle, mitochondrial pathway, Bax, natural compounds, anticancer therapy, polyphenols, intrinsic apoptosis

## Abstract

Cancer progression is closely associated with dysregulation of apoptosis, enabling malignant cells to evade programmed cell death and develop resistance to therapy. Among the key regulators of this process, the tumor suppressor protein p53 and the Bcl-2 family of proteins play central and interconnected roles in controlling cell survival and mitochondrial integrity. In recent years, naturally occurring flavonoids have attracted considerable attention as potential modulators of these pathways due to their diverse biological activities and relatively low toxicity. This review provides a focused and integrative overview of how different subclasses of flavonoids modulate the p53–Bcl-2 signaling axis to regulate apoptosis in cancer cells. Particular emphasis is placed on the mechanistic interplay between p53 stabilization, transcriptional regulation of apoptotic targets, mitochondrial outer membrane permeabilization, and caspase activation. In contrast to previous general reviews on flavonoids and cancer, this work provides an integrated overview of evidence across multiple flavonoid subclasses and experimental cancer models, highlighting both shared and pathway-specific apoptotic responses. Experimental findings from in vitro and in vivo studies are discussed, including the effects of quercetin, kaempferol, myricetin, epigallocatechin gallate, and related compounds on cell-cycle arrest, oxidative stress, mitochondrial dysfunction, and intrinsic apoptotic signaling. Furthermore, the review examines the relationship between flavonoid chemical structure and biological activity, with particular attention to bioavailability, metabolic transformation, and strategies aimed at improving therapeutic efficacy, including structural modification and nanocarrier-based delivery systems. Despite promising preclinical findings, significant translational challenges remain, including poor pharmacokinetic properties, variability among experimental models, and limited clinical validation. Overall, flavonoids represent a promising class of bioactive compounds capable of targeting apoptosis through modulation of the p53–Bcl-2 network, and a deeper mechanistic understanding of their activity may support the development of novel targeted and combination anticancer therapies.

## 1. Introduction

Cancer remains one of the most significant global health challenges of the 21st century, characterized by a steadily increasing incidence and persistently high mortality rates. According to the World Health Organization, approximately 20 million new cancer cases and nearly 10 million cancer-related deaths were reported worldwide in 2022. These numbers are expected to rise substantially, with projections estimating over 35 million new cases annually by 2050. Among the most prevalent malignancies are lung, colorectal, breast, and stomach cancers, while cervical cancer, primarily caused by persistent infection with high-risk human papillomavirus (HPV) types such as HPV16 and HPV18, remains a major contributor to global cancer burden.

At the molecular level, cancer is defined by uncontrolled cell proliferation resulting from the accumulation of genetic and epigenetic alterations that disrupt the regulation of the cell cycle. Under physiological conditions, cell cycle checkpoints ensure genomic integrity by preventing the propagation of damaged DNA. However, when these regulatory mechanisms fail, mutated cells can bypass checkpoint control, proliferate uncontrollably, and contribute to tumor development [1,2]. This process ultimately leads to progressive tissue dysfunction and, in advanced stages, organ failure and death.

Cancer development is influenced by a wide range of risk factors, including physical agents such as radiation, biological factors such as oncogenic viruses, and chemical carcinogens, most notably tobacco smoke. Lifestyle-related factors, including alcohol consumption and hormonal imbalances, further contribute to carcinogenesis. In addition, aging plays a crucial role, as the gradual accumulation of genetic mutations over time increases the likelihood of malignant transformation [3,4,5,6,7,8,9,10].

A hallmark of cancer progression is the disruption of apoptosis, a tightly regulated process responsible for maintaining cellular homeostasis by eliminating damaged or potentially harmful cells. In normal tissues, apoptosis acts as a protective mechanism, preventing the accumulation of genetically unstable cells. In contrast, cancer cells frequently acquire the ability to evade apoptosis through mutations in key regulatory genes, including the tumor suppressor p53 and members of the Bcl-2 protein family. This impairment of apoptotic signaling not only facilitates tumor initiation but also contributes to cancer progression, metastasis, and resistance to therapy [10].

Flavonoids are a large and structurally diverse group of naturally occurring polyphenolic compounds widely distributed in fruits, vegetables, medicinal plants, cereals, and beverages such as tea and wine. Increasing evidence indicates that flavonoids possess a broad spectrum of biological and pharmacological activities, including antioxidant, anti-inflammatory, antimicrobial, cardioprotective, neuroprotective, and anticancer effects. Their therapeutic potential is largely attributed to their ability to modulate oxidative stress, inflammation, mitochondrial function, cell-cycle progression, and multiple intracellular signaling pathways involved in cell survival and apoptosis. Recent studies have highlighted the importance of flavonoids not only as dietary bioactive compounds but also as promising candidates for pharmaceutical and nanomedicine-based applications due to their relatively low toxicity and multitarget mechanisms of action. In cancer research, flavonoids have attracted particular attention because of their capacity to regulate tumor cell proliferation, angiogenesis, metastasis, and apoptosis through modulation of signaling networks such as PI3K/Akt, NF-κB, MAPK, and p53-dependent pathways. Despite promising preclinical findings, important translational challenges remain, especially regarding bioavailability, metabolic stability, and targeted delivery, which has stimulated growing interest in structural modification and nanoformulation strategies [11,12,13,14].

Recent literature [15,16] has increasingly emphasized that the pro-apoptotic effects of flavonoids are strongly dependent on their structural features, including hydroxylation pattern, degree of conjugation, glycosylation, methylation, and the presence of lipophilic substituents. These structural determinants influence not only antioxidant capacity, cellular uptake, and bioavailability, but also the ability of flavonoids to interact with key signaling proteins involved in apoptosis. In particular, flavonols such as quercetin, kaempferol, and myricetin have been repeatedly associated with p53 stabilization or activation, increased Bax/Bcl-2 ratio, mitochondrial membrane depolarization, cytochrome c release, and downstream caspase activation. Recent comprehensive studies further indicate that flavonoids modulate additional cancer-relevant pathways, including PI3K/Akt/mTOR, NF-κB, MAPK, AMPK, and JAK/STAT signaling. In breast cancer models, flavonoid-mediated inhibition of JAK2/STAT3 has been linked to reduced expression of STAT3-regulated survival proteins, including Bcl-2, Mcl-1, survivin, VEGF, and cyclin D, together with activation of caspases-3, -7, and -9, thereby connecting flavonoid activity with apoptosis induction, mitochondrial signaling, and reversal of therapy resistance. Moreover, p53 should be considered not only as a classical apoptosis and cell-cycle regulator, but also as an important modulator of inflammation, immune surveillance, MHCI antigen presentation, TLR signaling, and immune checkpoint regulation. This broader perspective is particularly relevant because p53 dysfunction may contribute simultaneously to apoptosis evasion, chronic inflammation, immune escape, and therapeutic resistance. Therefore, current research increasingly supports a structure–mechanism–activity perspective, in which flavonoid-mediated apoptosis should be interpreted as coordinated modulation of mitochondrial, p53-related, inflammatory, and survival signaling networks rather than as a simple antioxidant or cytotoxic effect. Recent advances in structural modification and nanoformulation may further improve the bioavailability, stability, and tumor-targeting potential of flavonoids, thereby enhancing their translational relevance in apoptosis-based anticancer strategies.

Given the central role of apoptosis in cancer biology, significant attention has been directed toward identifying compounds capable of restoring or modulating apoptotic pathways. Among these, naturally occurring flavonoids have emerged as promising candidates due to their diverse biological activities, relatively low toxicity, and widespread presence in the human diet. The comprehensive review by Rahman et al. represents one of the most detailed analyses of flavonoid-mediated modulation of the p53/Bcl-2 signaling axis currently available, providing important mechanistic insight into apoptosis-oriented anticancer strategies [17].

The aim of this review is to provide a comprehensive overview of the molecular mechanisms by which flavonoids modulate apoptosis in cancer, with a particular focus on the p53–Bcl-2 regulatory axis. Special emphasis is placed on the ability of flavonoids to influence p53 activation and stability, regulate downstream apoptotic targets, and shift the balance between pro-apoptotic and anti-apoptotic members of the Bcl-2 family.

The literature included in this narrative review was identified through searches of PubMed, Scopus, and Web of Science databases. The search strategy focused on publications related to flavonoids, apoptosis, cancer, p53 signaling, Bcl-2 family proteins, mitochondrial dysfunction, oxidative stress, and anticancer activity. Representative keywords included “flavonoids”, “apoptosis”, “cancer”, “p53”, “TP53”, “Bcl-2”, “Bax”, “mitochondrial pathway”, “oxidative stress”, “cell death”, “nanoparticles”, “nanocarriers”, and combinations thereof.

The literature search primarily covered publications available up to May 2026. Original research articles, reviews, and relevant experimental studies investigating the effects of flavonoids on apoptosis-related signaling pathways were considered. Particular emphasis was placed on studies evaluating modulation of the p53–Bcl-2 axis, mitochondrial apoptotic signaling, oxidative stress responses, and translational aspects of flavonoid-based therapies.

Publications unrelated to cancer, apoptosis, or flavonoid-mediated signaling mechanisms were excluded unless they provided important mechanistic context. The final selection of references was based on scientific relevance, methodological quality, and direct contribution to understanding the role of flavonoids in regulating apoptosis and cancer-related signaling pathways.

The review is structured as follows: first, the fundamental mechanisms of apoptosis and the roles of p53 and Bcl-2 proteins are outlined. Next, the chemical structure, classification, and biological properties of flavonoids are discussed. This is followed by a detailed analysis of experimental evidence demonstrating the effects of selected flavonoids on apoptotic signaling pathways in various cancer models. Finally, the therapeutic potential of flavonoids is evaluated, with particular attention to current limitations, including bioavailability and translational challenges, as well as future perspectives for their application in cancer therapy.

## 2. Apoptosis and Its Central Regulators: The Role of p53 and Bcl-2 Proteins

### 2.1. The Role of Apoptosis

**Figure 1:**
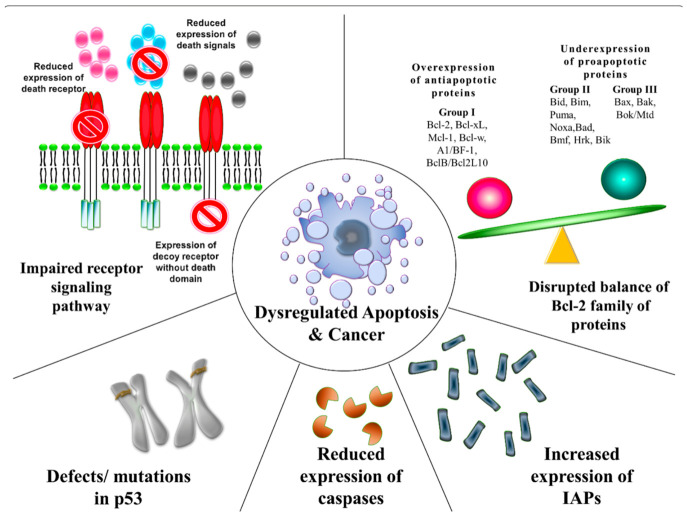
Dysregulated apoptosis and cancer: mechanisms of evasion of apoptosis. Major mechanisms underlying apoptosis dysregulation in cancer cells. The schematic illustrates how malignant cells evade programmed cell death through coordinated disruption of both extrinsic and intrinsic apoptotic signaling pathways. Impaired death receptor signaling, including reduced expression of death receptors, decreased availability of extracellular death ligands, and expression of nonfunctional decoy receptors, contributes to suppression of receptor-mediated apoptosis. Simultaneously, cancer progression is associated with defects or mutations in p53, leading to impaired DNA damage sensing, defective cell-cycle arrest, and reduced activation of pro-apoptotic signaling cascades. Dysregulation of the Bcl-2 family proteins further shifts the intracellular balance toward cell survival through overexpression of anti-apoptotic members (e.g., Bcl-2, Bcl-xL, Mcl-1) and reduced expression of pro-apoptotic proteins such as Bax, Bak, Bid, Puma, and Noxa. Additional resistance mechanisms include reduced caspase expression and increased levels of inhibitor of apoptosis proteins (IAPs), collectively suppressing apoptotic execution. These interconnected alterations promote cancer cell survival, therapeutic resistance, uncontrolled proliferation, and tumor progression by enabling evasion of apoptosis. Reprinted from [19], licensed under CC BY 2.0.

Apoptosis is a programmed process characterized by a series of morphological and biochemical changes in dying cells. These include cell shrinkage, loss of normal intercellular contacts, condensation of chromatin into dense masses, formation of membrane blebs and apoptotic bodies, followed by rapid phagocytosis by professional phagocytes or neighboring cells [18] (Figure 1, Ref. [19]).

From the earliest stages of life, apoptosis serves as a sculptor of form, guiding the development of organs and limbs. It contributes to the proper shaping of organs and the removal of interdigital tissue, forming distinct fingers and toes. Disruptions in apoptotic mechanisms can lead to fetal abnormalities; for instance, studies have shown that wild-type p53 mouse embryos undergo miscarriages following radiation-induced teratogenesis, whereas p53-deficient embryos do not. Both the nervous and immune systems depend on apoptosis during development. Initially, these systems produce an excess of cells, and apoptosis selectively eliminates those that fail to establish effective synaptic connections or functional antigen-specificities. Such large-scale but precisely controlled cell elimination requires tight regulation. In adult organisms, approximately ten billion cells undergo apoptosis each day to maintain equilibrium with newly formed cells derived from stem cell populations. This balance of cell turnover represents an active, regulated process rather than a passive one. The same apoptotic mechanisms are responsible for clearing damaged or defective cells. With advancing age, the regulation of apoptosis in response to DNA damage may become dysregulated. Overactivation of apoptotic pathways can contribute to degenerative diseases, while reduced sensitivity to apoptotic signals may increase the risk of cancer development [20,21,22].

Apoptosis was first discovered by Carl Vogt in 1848, providing a new understanding of how old cells are replaced by new ones. The fate of a cell, including its survival or death, is genetically regulated. Initial research on apoptosis was conducted using the nematode *Caenorhabditis elegans*, where it was demonstrated that the process is directly governed by three specific genes. Among them, ced-3 and ced-4 act as executioner genes, promoting cell death, while ced-9 functions as a protective gene that prevents apoptosis. Ced-9 inhibits ced-3 activity with the assistance of ced-4, which acts as a mediator or bridge between the two. These genes have been identified as homologous to the genes coding for the Bcl-2 family of apoptosis regulators in vertebrates [23,24]. Nearly twenty years later, in his Lectures on Cellular Pathology, Rudolph Virchow distinguished between two forms of cell death: necrosis and necrobiosis. Using the term “necrobiosis,” he morphologically described apoptosis as a degenerative process in which tissue softens, yet the overall structure of the tissue remains intact until it is eventually completely lost [25].

Apoptosis was originally characterized in 1972 as a mechanism of programmed cell elimination that maintains the balance of cell proliferation; however, related processes had been described under various terms since the mid-19th century. Since the early 1990s, the concept of apoptosis has occasionally been contested, redefined, or inconsistently applied. To maintain conceptual clarity, this work adheres to the original definition by Kerr et al. (1972) and the subsequent clarifications provided by the Nomenclature Committee on Cell Death in 2009 [26,27].

Two primary apoptosis pathways have been identified: the intrinsic, or mitochondrial, pathway, which is regulated by Bcl-2 family proteins, and the extrinsic, or death-receptor, pathway, which is triggered by the interaction between Fas and its ligand, FasL.

### 2.2. Intrinsic Pathway of Apoptosis

The intrinsic pathway, also known as the mitochondrial pathway, operates under precise regulatory control, responding to diverse forms of cellular stress that promote the release of pro-apoptotic molecules. A finely tuned equilibrium between pro- and anti-apoptotic members of the Bcl-2 family is essential for preserving mitochondrial integrity and determining whether a cell survives or undergoes death [28]. A key regulatory component of the mitochondria-mediated apoptotic pathway is the Bcl-2 protein family [29]. This family comprises pro-apoptotic members, including Bax, Bak, Bik, Bad, and Bid, as well as anti-apoptotic proteins such as Bcl-2 and Bcl-xL, which are classified based on their capacity to either promote or inhibit the release of cytochrome c [30,31] (Table 1).

**Figure 2:**
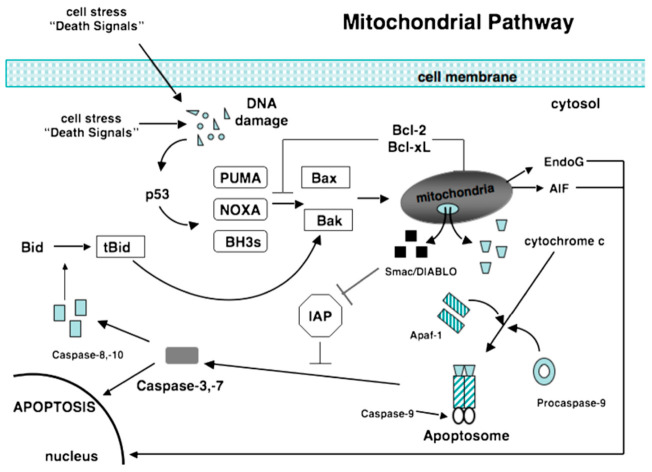
Mitochondrial (intrinsic) apoptotic pathway regulated by p53 and Bcl-2 family proteins. Cellular stress activates p53-dependent signaling, leading to modulation of Bcl-2 family proteins, mitochondrial outer membrane permeabilization (MOMP), cytochrome c release, apoptosome formation, and activation of caspase-dependent apoptosis. Crosstalk with the extrinsic pathway through Bid cleavage is also indicated. Reprinted with permission from [35].

Activation of the intrinsic apoptotic pathway in response to diverse stress stimuli leads to Mitochondrial Outer Membrane Permeabilization (MOMP) (Figure 2) [27]. MOMP causes the release of pro-apoptotic proteins from the intermembrane space, which facilitates the assembly of the apoptosome, subsequently activating caspase-9 and triggering downstream caspases-3 and -7 [32,33]. Within the mitochondrial pathway, caspase-9 serves as the primary initiator caspase [34]. Mitochondrial dysfunction is a defining feature of the intrinsic apoptotic pathway and is frequently observed following flavonoid treatment in cancer cells. One of the earliest events is the disruption of mitochondrial membrane potential (ΔΨm), which reflects loss of mitochondrial integrity and impaired bioenergetic function. Depolarization of the mitochondrial membrane is commonly accompanied by activation of Bax and Bak, leading to mitochondrial outer membrane permeabilization (MOMP). As a consequence, cytochrome c and other pro-apoptotic factors are released from the intermembrane space into the cytosol. Cytosolic cytochrome c subsequently associates with apoptotic protease activating factor-1 (Apaf-1) and procaspase-9 to form the apoptosome, resulting in activation of caspase-9 and downstream executioner caspases, including caspase-3 and caspase-7. Numerous flavonoids have been reported to induce mitochondrial membrane depolarization and cytochrome c release, indicating that mitochondrial dysfunction represents a major mechanism contributing to their pro-apoptotic activity.

Within the Bcl-2 family, Bax and Bak function as the principal executioners of mitochondrial apoptosis. Upon activation, these proteins undergo conformational changes and oligomerization within the outer mitochondrial membrane, leading to mitochondrial outer membrane permeabilization (MOMP) and release of cytochrome c. In contrast, anti-apoptotic proteins such as Bcl-2, Bcl-xL, and Mcl-1 preserve mitochondrial integrity by sequestering pro-apoptotic Bcl-2 family members and preventing their activation. Consequently, the relative balance between pro-apoptotic and anti-apoptotic proteins represents a critical determinant of cellular susceptibility to apoptosis. Numerous flavonoids have been reported to increase Bax and Bak expression while simultaneously reducing levels of Bcl-2, Bcl-xL, and Mcl-1, thereby shifting the cellular equilibrium toward mitochondrial dysfunction, caspase activation, and apoptotic cell death.

### 2.3. Extrinsic Pathway of Apoptosis

**Figure 3:**
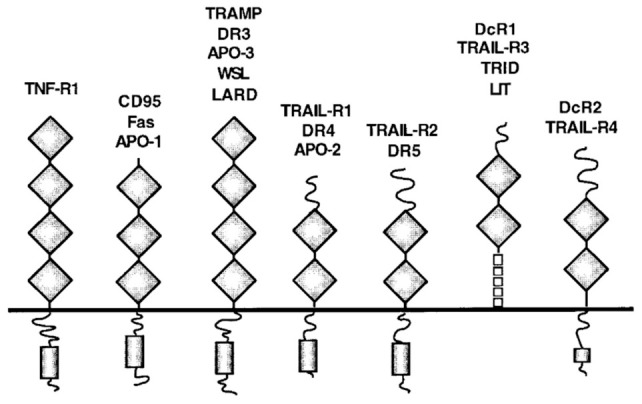
Members of the TNF receptor superfamily involved in extrinsic apoptotic signaling. Activation of death receptors by their corresponding ligands initiates DISC formation, caspase activation, and apoptotic signaling. The figure also illustrates the role of decoy receptors and receptor balance in determining cellular susceptibility to apoptosis. Reprinted with permission from [37].

The interaction between extracellular death ligands and membrane-bound death receptors serves as the initial step of the extrinsic apoptotic cascade. The extrinsic, or death receptor, pathway of apoptosis involves specific TNF receptor family members, including DR3, Fas, TNF-R1, TRAIL-R1, and TRAIL-R2 (Figure 3) [36].

The pathway is triggered when specific death ligands interact with their corresponding death receptors. Among the several identified receptors, the most well-characterized are the type 1 TNF receptor (TNFR1) and Fas (CD95), whose ligands are TNF and Fas Ligand (FasL) [38,39,40]. These receptors contain an intracellular death domain that recruits adaptor proteins, including TNF receptor-associated death domain (TRADD) and Fas-associated death domain (FADD), along with cysteine proteases such as caspase-8 [41]. Ligand binding to the receptor facilitates the formation of a receptor-adaptor complex known as the Death-Inducing Signaling Complex (DISC). DISC then promotes the assembly and activation of pro-caspase-8 [35]. Once activated, caspase-8 functions as an initiator caspase, triggering apoptosis through the cleavage and activation of downstream effector caspases [19].

**Figure 4:**
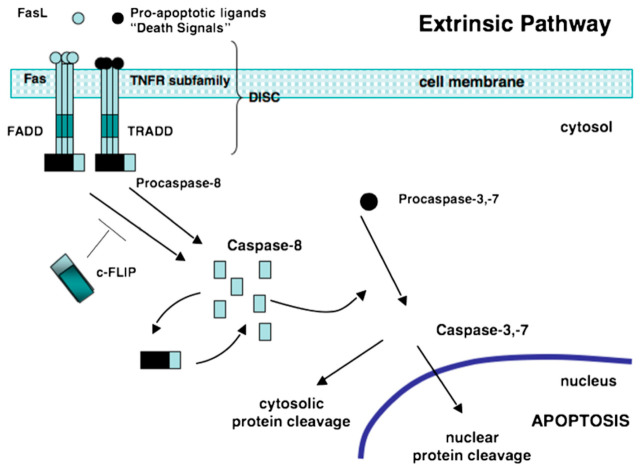
Extrinsic (death receptor-mediated) apoptotic pathway. Binding of extracellular death ligands to death receptors promotes DISC assembly, activation of initiator caspases, and subsequent activation of effector caspases leading to apoptosis. The figure also illustrates inhibition by c-FLIP and communication with the intrinsic pathway through Bid cleavage. Reprinted with permission from [35].

The extrinsic apoptotic pathway (Figure 4) plays a vital role in eliminating cells that are infected or have the potential to become cancerous (Table 2). In the context of viral infections, this pathway is crucial for removing cells that harbor viruses. While antibodies can neutralize viruses outside of cells and prevent infection by inhaled viruses, they are less effective against viruses that have already entered host cells and established an infection [42].

### 2.4. Cross-Talk Between Intrinsic and Extrinsic Apoptotic Pathways

Although flavonoid-mediated apoptosis is frequently discussed in the context of the intrinsic mitochondrial pathway, increasing evidence indicates substantial cross-talk between intrinsic and extrinsic apoptotic signaling. In many cancer models, flavonoids regulate apoptosis through coordinated activation of death receptor-associated mechanisms together with mitochondrial dysfunction and p53-dependent signaling. Activation of extrinsic pathways involving Fas/FasL, TRAIL receptors, and caspase-8 may amplify mitochondrial apoptosis through cleavage of BID into truncated BID (tBID), which subsequently promotes mitochondrial outer membrane permeabilization, cytochrome c release, and activation of downstream caspase cascades.

At the same time, flavonoid-induced ROS accumulation and p53 activation may sensitize cancer cells to death receptor-mediated apoptosis by altering the Bax/Bcl-2 balance and reducing anti-apoptotic signaling. This bidirectional interaction between intrinsic and extrinsic pathways is particularly important because it may enhance apoptotic efficiency and partially overcome resistance mechanisms commonly observed in cancer cells. Therefore, flavonoid-mediated apoptosis should be interpreted as an integrated signaling process involving dynamic communication between mitochondrial stress responses, death receptor activation, redox regulation, and caspase-dependent execution pathways rather than as activation of isolated apoptotic modules.

Although flavonoids are traditionally recognized for their antioxidant properties, increasing evidence suggests that their biological effects on oxidative stress are highly context-dependent. In normal cells, flavonoids often act as ROS scavengers and protect against oxidative damage. In contrast, in cancer cells, many flavonoids exhibit pro-oxidant activity, promoting ROS accumulation beyond the cellular antioxidant capacity. This increase in oxidative stress may trigger mitochondrial membrane depolarization, DNA damage responses, p53 activation, cytochrome c release, and subsequent caspase-dependent apoptosis. Therefore, the anticancer activity of flavonoids frequently relies not only on antioxidant protection but also on their ability to induce selective oxidative stress in tumor cells.

### 2.5. The Difference Between Apoptosis and Necrosis

In most standard texts, cellular death is characterized as coagulative necrosis, which appears unrelated to mechanisms that govern cell population control [26]. The term *necrosis* originates from the Greek *nekros* (“dead” or “corpse”) and -*osis* (“process”), denoting a state of degeneration or decay [44,45]. The 1998 study that first examined necroptosis did not describe its features in detail. Later, key research found that necrosis lacks chromatin condensation and apoptotic body formation but involves swelling of mitochondria and other organelles, though the specific organelles forming cytoplasmic vacuoles were not determined [46,47].

Necrosis differs from apoptosis in almost every aspect, representing a distinct pathway of cell demise [48]. In contrast to apoptosis, necrosis is viewed as a detrimental process in which the cell plays a passive role, undergoing death through an energy-independent mechanism [49]. Necrosis may be triggered by bacterial toxins [50] and by elements of the immune response, including complement proteins [51], activated Natural Killer cells [52], and peritoneal macrophages [53,54].

Despite distinct mechanisms and morphological characteristics, apoptosis and necrosis share overlapping features. Research suggests that both processes are manifestations of a common biochemical pathway, often referred to as the “apoptosis-necrosis continuum” [49]. However, apoptosis is preferred due to its features. In anticancer therapy, targeting apoptosis is considered safer than inducing necrosis due to the highly regulated and non-inflammatory nature of apoptotic cell death. Apoptosis allows for the controlled elimination of malignant cells without the release of intracellular contents, thereby minimizing tissue damage and preventing the activation of a systemic inflammatory response. In contrast, necrosis is an uncontrolled process associated with cell lysis and the release of endogenous molecules, which can promote inflammation, tumor progression, and adverse clinical outcomes [49].

### 2.6. The p53 Pathway

The p53 protein is recognized as one of the key regulators of the cell cycle, as it becomes stabilized and activated in response to various types of cellular stress. These include ionizing radiation, electromagnetic radiation (like ultraviolet light), biological stressors (such as viral infections or bacterial toxins), chemical stressors (toxic substances), endogenous stress (including excessive production of reactive oxygen species, ROS, which can severely damage cellular components), hypoxia, and uncontrolled oncogene activation. In response to these DNA-damaging conditions, p53 can initiate multiple biological responses [55,56,57]. It consists of five major structural regions: the transactivation domain, the proline-rich domain, the DNA-binding domain, the tetramerization domain, and the regulatory domain [57].

Under normal circumstances, the cellular levels of p53 protein remain very low because its negative regulators, MDM2 and MDMX, tightly control it by promoting its degradation through ubiquitination [58]. The MDM2 protein possesses several structural features essential for suppressing p53 activity. It directly binds to p53 via its N-terminal domain, which creates a hydrophobic pocket that accommodates a helical segment of p53’s transactivation domain [59,60]. Both proteins interact with p53, with MDM2 functioning as an E3 ubiquitin ligase and Mdmx serving as a co-factor that enhances MDM2’s activity. This interaction promotes p53 degradation and suppresses the transcription of p53 target genes, including p21, Gadd45a, and various DNA-repair enzymes. When p53-mediated processes like cell-cycle arrest, senescence, and apoptosis are lost, the cellular “gatekeeper” that prevents the proliferation of damaged chromosomes is removed. As a result, overexpression of Mdm2 or MDMX can lead to centrosome amplification, defective mitotic spindles, and aneuploidy [61].

The p53 protein is subject to numerous post-translational modifications (PTMs), such as ubiquitination, phosphorylation, acetylation, methylation, SUMOylation, NEDDylation, O-GlcNAcylation, ADP-ribosylation, UFMylation, hydroxylation, β-hydroxybutyrylation, sulfation, and isoLG adduction [62]. The phosphorylation cascade acts as an upstream regulator of the p53-MDM2 feedback loop. In response to cellular stressors such as DNA damage, hypoxia, or oncogene activation, kinases, including DNA-PK, ATM, CK1, CK2, and JNK, phosphorylate specific serine residues within the N-terminal region of p53 (such as Ser-15, Ser-33, and Ser-37). These phosphorylation events trigger conformational changes that significantly reduce p53’s binding affinity for its negative regulator, MDM2. As a result, MDM2-mediated ubiquitination and repression of p53 are inhibited, allowing p53 to accumulate, stabilize, and activate the transcription of its target genes [63].

An important factor influencing the anticancer activity of flavonoids is the p53 status of the investigated cancer model. In tumors expressing wild-type p53, many flavonoids promote apoptosis through direct or indirect stabilization and activation of p53, leading to transcriptional upregulation of pro-apoptotic mediators such as Bax, PUMA, NOXA, and p21, together with suppression of anti-apoptotic proteins including Bcl-2 and Bcl-xL. These events facilitate mitochondrial outer membrane permeabilization, cytochrome c release, and caspase-dependent apoptosis.

However, several studies also demonstrate substantial flavonoid activity in mutant or p53-deficient cancer models, indicating the existence of partially p53-independent mechanisms. In such contexts, flavonoids may induce apoptosis through excessive ROS generation, mitochondrial dysfunction, disruption of cellular redox homeostasis, direct modulation of Bcl-2 family proteins, activation of stress-related kinases, or inhibition of survival pathways such as PI3K/Akt, NF-κB, MAPK, and STAT3 signaling. Therefore, the biological effects of flavonoids should not be interpreted exclusively as classical p53-reactivating events, but rather as multifactorial modulation of interconnected apoptotic and stress-response networks, whose relative contribution depends on the molecular background and p53 functional status of the tumor cells.

Activation of p53 represents a critical molecular link between cellular stress sensing and execution of the intrinsic apoptotic pathway. Following stabilization, p53 promotes transcription of pro-apoptotic proteins such as Bax, PUMA, and NOXA while suppressing anti-apoptotic regulators including Bcl-2 and Bcl-xL. The resulting shift in the balance of Bcl-2 family proteins facilitates mitochondrial outer membrane permeabilization (MOMP), leading to cytochrome c release into the cytosol. Cytochrome c subsequently associates with Apaf-1 and procaspase-9 to form the apoptosome, triggering activation of caspase-9 and downstream executioner caspases. Therefore, p53, Bcl-2 family proteins, MOMP, and caspase activation constitute a tightly interconnected signaling network that ultimately determines cellular susceptibility to apoptosis.

Several flavonoids have been reported to influence upstream regulators of p53 rather than acting exclusively on downstream apoptotic targets. In particular, flavonoid-induced oxidative stress and DNA damage may activate ATM/ATR-dependent signaling pathways, leading to phosphorylation and stabilization of p53. Furthermore, inhibition of MDM2-mediated p53 degradation has been proposed as an additional mechanism contributing to p53 accumulation and activation. Through modulation of these upstream regulatory networks, flavonoids may indirectly enhance p53-dependent transcriptional responses, resulting in increased expression of pro-apoptotic mediators and activation of mitochondrial apoptosis.

### 2.7. Flavonoid-Induced Apoptosis in p53-Deficient and p53-Mutant Cancers

Although activation of wild-type p53 represents one of the most frequently reported mechanisms underlying flavonoid-induced apoptosis, accumulating evidence indicates that many flavonoids retain significant anticancer activity in p53-mutant or p53-deficient cancer models (Table 3). This observation is particularly important because TP53 mutations occur in approximately half of all human cancers and are often associated with poor prognosis, resistance to therapy, and impaired apoptotic responses.

In p53-deficient contexts, flavonoids may induce apoptosis through alternative mechanisms that bypass the requirement for functional p53 signaling. One of the most commonly reported mechanisms involves excessive generation of reactive oxygen species (ROS), resulting in oxidative stress, mitochondrial dysfunction, disruption of mitochondrial membrane potential, and subsequent release of cytochrome c. These events ultimately promote activation of initiator and effector caspases and trigger apoptotic cell death.

In addition, numerous flavonoids have been shown to suppress pro-survival signaling pathways, particularly NF-κB. Constitutive activation of NF-κB is frequently observed in cancer cells and contributes to resistance against apoptosis through increased expression of anti-apoptotic proteins such as Bcl-2, Bcl-xL, survivin, and Mcl-1. By inhibiting NF-κB signaling, flavonoids may restore apoptotic sensitivity even in the absence of functional p53.

Another important mechanism involves direct modulation of Bcl-2 family proteins. Several flavonoids have been reported to increase expression of pro-apoptotic proteins such as Bax and Bak while simultaneously reducing levels of anti-apoptotic proteins, including Bcl-2, Bcl-xL, and Mcl-1. This shift in the Bax/Bcl-2 balance promotes mitochondrial outer membrane permeabilization, cytochrome c release, and activation of the intrinsic apoptotic pathway independently of p53 transcriptional activity.

Importantly, TP53 mutations should not be considered a uniform category. Different TP53 alterations may produce distinct biological outcomes, including loss-of-function (LOF), dominant-negative (DN), and gain-of-function (GOF) phenotypes. Loss-of-function mutations impair the ability of p53 to regulate cell-cycle arrest and apoptosis, whereas dominant-negative mutants may interfere with the activity of residual wild-type p53. In contrast, gain-of-function mutations can actively promote tumor progression, survival signaling, metastasis, and therapeutic resistance. These molecular differences may substantially influence responsiveness to flavonoids. In tumors harboring LOF or DN mutations, apoptosis induction may depend primarily on p53-independent mechanisms such as ROS accumulation, mitochondrial dysfunction, and direct modulation of Bcl-2 family proteins. Conversely, in tumors expressing GOF mutants, flavonoids may exert additional effects by suppressing oncogenic signaling pathways associated with mutant p53. Therefore, interpretation of flavonoid-mediated apoptosis should consider not only TP53 mutational status but also the functional consequences of specific TP53 alterations.

Collectively, these findings indicate that flavonoid-mediated apoptosis should not be viewed exclusively as a consequence of p53 activation. Instead, flavonoids appear capable of targeting multiple interconnected signaling networks, allowing them to retain anticancer activity even in tumors characterized by TP53 mutations or complete loss of p53 function.

Although the p53–Bcl-2 axis represents one of the most extensively studied mechanisms underlying flavonoid-induced apoptosis, available evidence indicates that it should not be considered the sole or universally dominant pathway. Many flavonoids retain significant anticancer activity in TP53-mutant and TP53-null models, suggesting the involvement of alternative signaling mechanisms. Depending on the flavonoid structure, cellular context, and genetic background of the tumor, apoptosis may be driven by ROS accumulation, mitochondrial dysfunction, NF-κB suppression, modulation of survival pathways, or direct regulation of Bcl-2 family proteins independently of p53. Therefore, the p53–Bcl-2 axis should be viewed as an important component of a broader signaling network rather than a universally applicable mechanism of flavonoid action.

## 3. Flavonoids: Structure, Bioactivity, and Therapeutic Potential

Although flavonoids are commonly classified according to their chemical subclasses, increasing evidence suggests that their anticancer effects can also be grouped according to dominant apoptotic mechanisms. Across different flavonoid families, several recurring mechanistic patterns can be identified, including stabilization and activation of p53, modulation of the Bax/Bcl-2 ratio, ROS-dependent mitochondrial dysfunction, cytochrome c release, and activation of caspase cascades. Importantly, individual flavonoids often affect multiple interconnected signaling pathways simultaneously, linking oxidative stress, mitochondrial integrity, inflammatory signaling, and cell-cycle regulation. Therefore, the following sections discuss flavonoids not only according to structural subclass, but also in the context of their predominant mechanisms of apoptosis induction and p53–Bcl-2 pathway modulation.

### 3.1. Structure

Flavonoids represent a significant group of natural compounds and are classified as plant secondary metabolites with polyphenolic structures. They are commonly present in a wide variety of fruits, vegetables, and some beverages [64]. They are produced through the phenylpropanoid pathway. Existing studies suggest that phenolic secondary metabolites, such as flavonoids, play a key role in a wide range of pharmacological activities [65]. Their biological activities are influenced by their molecular structure.

The biological activity of flavonoids is strongly influenced by their chemical structure. The chemical properties of flavonoids depend on several structural factors such as their structural class, level of hydroxylation, types of substitutions and conjugations, as well as the extent of polymerization [66]. Flavonoids possess a characteristic C6-C3-C6 carbon skeleton, yet differences in ring saturation and the presence of hydroxyl, methoxy, or sugar groups give rise to distinct subclasses, such as flavones, flavonols, and flavanones, and significantly influence both their absorption and their biological activities in humans [67]. Glycosylation represents the predominant modification of flavonoids in plants. The attachment of sugar moieties usually enhances their solubility and facilitates membrane transport, but limits their bioavailability. However, only aglycones and small glucosides can efficiently pass through the intestinal epithelium. Larger glycosides require enzymatic deglycosylation prior to absorption. As a result, flavonoids occurring mainly as aglycones, such as quercetin and kaempferol, exhibit greater bioavailability compared to highly glycosylated compounds like anthocyanins, which are absorbed less efficiently [68]. Glycosylated forms undergo biotransformation by gut microbiota, allowing them to be absorbed. Intestinal flora includes *Bifidobacterium adolescentis*, *Bifidobacterium longum*, *Enterococcus faecalis*, *Bacteroides ovatus*, *Bacteroides uniformis*, and *Parabacteroides distasonis* that biotransforms flavonoids through active enzymes like β-glucuronidase and α-L-Rhamnosidases [69].

The quantity and arrangement of hydroxyl groups on the B-ring, particularly the catechol configuration, and on the C-ring largely determine a flavonoid’s antioxidant strength, as these groups are capable of donating electrons and stabilizing free radical intermediates [70]. Overall, variations in flavonoid structure underpin their bioavailability and account for the wide range of antioxidant, anti-inflammatory, and anticancer effects observed in humans.

Several structural motifs have been associated with enhanced pro-apoptotic activity of flavonoids. In particular, the presence of a catechol moiety (3′,4′-dihydroxylation) within the B-ring has been linked to increased redox activity, ROS modulation, and activation of apoptosis-related signaling pathways. The C2=C3 double bond conjugated with the 4-oxo group promotes molecular planarity and facilitates interactions with intracellular targets involved in cell-cycle regulation and apoptosis. Increased hydroxylation, particularly in flavonols such as quercetin and myricetin, has frequently been associated with enhanced p53 activation, increased Bax/Bcl-2 ratio, and mitochondrial apoptotic signaling. Furthermore, gallate-containing structures, exemplified by epigallocatechin gallate (EGCG), may strengthen interactions with proteins regulating oxidative stress and apoptosis, contributing to enhanced modulation of Bcl-2 family proteins and caspase activation. Collectively, these observations indicate that specific structural motifs play an important role in determining the ability of flavonoids to regulate the p53–Bcl-2 axis and induce apoptosis in cancer cells.

A mechanistic structure–activity perspective suggests that several key structural features contribute to the ability of flavonoids to modulate apoptosis-related signaling pathways. Increased hydroxylation, particularly within the B-ring catechol motif, is frequently associated with enhanced redox activity, ROS modulation, p53 activation, and increased Bax/Bcl-2 ratios. The presence of a C2=C3 double bond conjugated with a 4-oxo group promotes molecular planarity and may facilitate interactions with intracellular signaling proteins involved in apoptosis regulation. Methoxylation generally increases lipophilicity and membrane permeability, potentially improving intracellular accumulation, although excessive methoxylation may reduce antioxidant capacity. In contrast, glycosylation often decreases direct biological activity due to reduced cellular uptake but may improve solubility and influence metabolic stability. Consequently, apoptosis induction by flavonoids appears to result from a complex interplay between structural determinants governing cellular availability, redox behavior, p53 responsiveness, mitochondrial signaling, and regulation of Bcl-2 family proteins (Table 4).

### 3.2. Classification

Flavonoids can be categorized into several classes, including flavones (such as flavone, apigenin, and luteolin), flavonols (such as quercetin, kaempferol, myricetin, and fisetin), and flavanones (such as flavanone, hesperetin, and naringenin), among others (Table 5) [71].

#### 3.2.1. Flavones

Flavones, a major subclass of flavonoids, are characterized by a 4H-chromen-4-one structure with a phenyl group attached at the 2-position. They are commonly found in nature as 7-O-glycoside derivatives [72]. Flavones share the basic flavonoid structure but possess distinct features that enhance their anti-inflammatory activity. The presence of a C2–C3 double bond keeps the A and B-rings coplanar, allowing greater conjugation and stronger interactions with inflammatory targets. Additionally, hydroxyl groups at the C5 and C4′ positions are crucial for inhibiting mediators such as NO, TNF-α, and COX-2. These structural traits give flavones, such as apigenin and chrysin, significantly higher potency than related flavanones, making them more effective in reducing inflammation at much lower concentrations [73,74].

#### 3.2.2. Flavonols

Flavonols, also known as 3-hydroxyflavones, are characterized by distinctive substitutions on their A and B rings, which are linked by a three-carbon bridge [65]. They usually contain hydroxyl groups at positions 5 and 7 on the A-ring, which contribute to their chemical reactivity and biological activity [72,75]. Flavonols exhibit a wide range of biological activities, including antioxidant [76], antibacterial, cardioprotective [77], anticancer [78], and antiviral effects [74,79]. Common examples of flavonols include quercetin, galangin, kaempferol, and myricetin [80].

#### 3.2.3. Flavanones

Flavanones, also known as dihydroflavones, are a class of flavonoids distinguished by their fully saturated C-ring structure. The lack of a double bond between the 2nd and 3rd carbon atoms in the C-ring is the primary feature that differentiates them from other flavonoid subclasses [81]. Typically, flavanones have hydroxyl groups attached to the 5 and 7 positions of the A-ring, and they may also contain hydroxyl or methoxy groups at the C3 or C4 positions on the B-ring [82]. Among flavanones, naringenin has attracted considerable attention due to its anticancer potential. Experimental studies have demonstrated that naringenin can inhibit tumor cell proliferation, induce cell-cycle arrest, promote apoptosis, and modulate oxidative stress and inflammatory signaling pathways associated with cancer progression. These biological activities are believed to contribute to its ability to regulate multiple molecular targets involved in tumor development and apoptosis [83,84,85,86].

#### 3.2.4. Isoflavones

Isoflavones are distinguished from other flavonoids by the placement of their B-ring at the C3 position of the heterocyclic C-ring within the diphenylpropane (C6–C3–C6) framework, marking their sole structural difference [84]. Flavonoids, including isoflavones and their metabolites, have been shown to trigger apoptosis in human gastric cancer-derived cells [87]. In addition to their pro-apoptotic activity, isoflavones have been reported to modulate signaling pathways involved in cell proliferation, inflammation, and tumor progression [88]. Among naturally occurring isoflavones, genistein is one of the most extensively investigated compounds and has been shown to influence multiple molecular targets associated with apoptosis and cancer cell survival, partly through modulation of tyrosine kinase-dependent signaling pathways [88,89]. Soybeans and their derived products provide the main isoflavones in the human diet, chiefly daidzein and genistein. After consumption, these compounds can display both estrogen-like and anti-estrogenic activities [90].

#### 3.2.5. Anthocyanins

Anthocyanins are naturally occurring pigments that dissolve in water and belong to the broader flavonoid family. Chemically, they are glycosidic compounds derived from polyhydroxy or polymethoxy forms of 2-phenylbenzopyrylium salts. Each anthocyanin molecule is composed of an aglycone portion, known as anthocyanidin, bound to a sugar unit such as glucose, xylose, galactose, arabinose, rhamnose, or rutinose. The sugar component is usually connected to the anthocyanidin framework through the hydroxyl group located at the C3 position of the C-ring [91,92]. Anthocyanins exhibit potent antioxidant activity and have been reported to modulate several signaling pathways associated with oxidative stress, inflammation, cell survival, and apoptosis. Through regulation of mediators such as cyclooxygenase-2 (COX-2), NF-κB, and pro-inflammatory cytokines, anthocyanins may contribute to the suppression of tumor-promoting inflammatory processes and the induction of apoptosis in cancer cells [93,94].

#### 3.2.6. Flavanols

Flavanols, commonly referred to as catechins or flavan-3-ols, possess a hydroxyl substituent on the third carbon of the C-ring and are defined by the absence of a 2,3-double bond within that ring structure [72,95]. This subclass includes biologically active compounds such as catechin, epicatechin, epigallocatechin, and epigallocatechin gallate (EGCG), many of which have attracted considerable interest because of their anticancer properties. Flavanols have been shown to regulate oxidative stress, mitochondrial function, cell-cycle progression, and apoptosis-related signaling pathways. In particular, EGCG has been extensively investigated for its ability to modulate p53 activity, alter the Bax/Bcl-2 balance, promote caspase activation, and suppress pro-survival signaling pathways in various cancer models [96,97].

#### 3.2.7. Chalcones

Chalcones are characterized by a 1,3-diaryl-2-propen-1-one framework, consisting of two aromatic rings (A and B) connected by a three-carbon α,β-unsaturated carbonyl system [98]. Preclinical research on chalcones and their derivatives has demonstrated strong potential for these compounds to act as antidiabetic, anticancer, anti-inflammatory, antimicrobial, antioxidant, antiparasitic, psychoactive, and neuroprotective agents [99].

**Figure 5:**
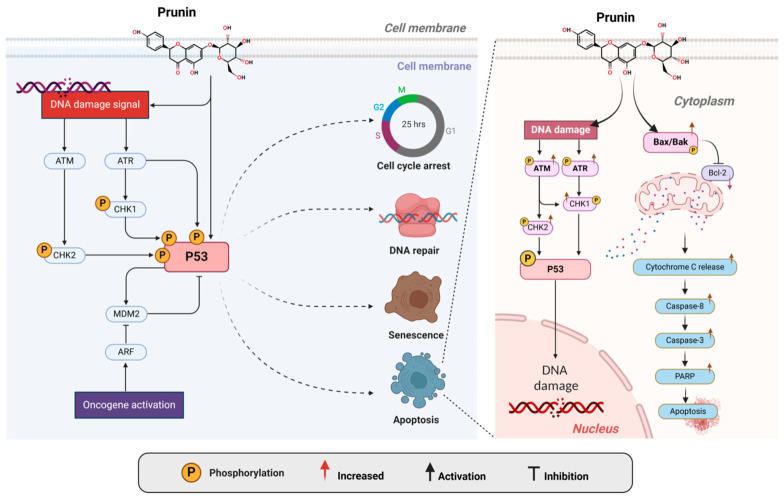
The prunin compound induces activation of P53 pathways. The prunin stimulates the P53 pathways after receiving the DNA damage signal, which further leads to multiple tasks, including cell cycle arrest, DNA repair if the damage is moderate, senescence, and apoptosis [101,102,103]. The apoptosis mechanism is highlighted when DNA damage stimulation occurs by prunin. It increases the activation of ATM, ATR, CHK1, and CHK2, which further leads to the activation of its downstream marker P53. The P53 further activates BAX and caspase cascades to induce apoptosis in cancer cells [58]. The figure was prepared using Biorender. Figure and its caption reprinted from [100], licensed under CC BY 4.0.

Recent studies have identified additional flavonoids with significant apoptosis-modulating potential, including prunin and isorhamnetin. Prunin, a flavanone glycoside structurally related to naringin, has recently emerged as a promising anticancer compound capable of influencing multiple pathways associated with cell-cycle regulation, oxidative stress, mitochondrial apoptosis, and tumor progression. Experimental evidence suggests that prunin can induce G_1_/S and G_2_/M cell-cycle arrest through modulation of cyclins and cyclin-dependent kinases, while simultaneously promoting mitochondrial apoptosis via increased Bax/Bak signaling, cytochrome c release, caspase-3/-9 activation, and ROS-dependent mechanisms. Additionally, prunin has been associated with suppression of VEGF-mediated angiogenesis and inhibition of pro-survival signaling pathways, including NF-κB, MAPK, and STAT-related pathways (Figure 5) [100].

**Figure 6:**
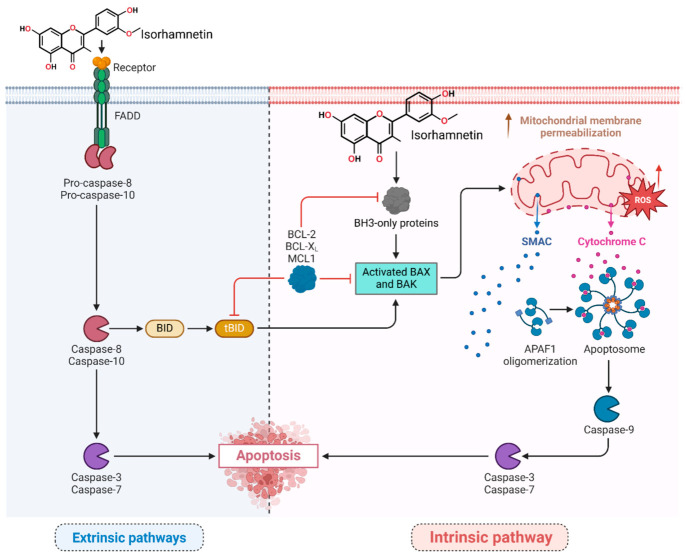
The isorhamnetin compound induces apoptosis in numerous tumor cells by triggering intrinsic and extrinsic-mediated pathways. In the extrinsic pathway, the isorhamnetin compound triggers the FAS receptor, activates FADD, and cleaves pro-caspase-8/10. Stimulated caspase-8 then processes BID into tBID, which links the extrinsic pathway to the intrinsic pathway by increasing the permeabilization of the outer membrane of mitochondria. In the intrinsic pathway, isorhamnetin induced mitochondrial dysfunction by increasing endogenous ROS levels and disrupting the balance (upregulating) between pro-apoptotic (BAX, BAK) and (downregulating) anti-apoptotic (BCL-XL, BCL-2, MCL1) proteins. This scenario leads to cytochrome c and SMAC release from mitochondria. Cytochrome c forms the apoptosome with the APAF1 marker, leading to the activation of caspase-9. Both pathways meet to activate effector caspases (caspase-3 and caspase-7) and induce apoptosis by isorhamnetin [105,106,107,108]. The figure was prepared using Biorender. The figure and its caption were reprinted from [104], licensed under CC BY 4.0.

Similarly, isorhamnetin, a methylated flavonol and structural derivative of quercetin, has gained increasing attention due to its broad anticancer activity and relatively favorable pharmacological profile. Recent reports indicate that isorhamnetin can regulate apoptosis through modulation of the Bax/Bcl-2 balance, mitochondrial membrane permeability, caspase activation, ROS generation, and p53-associated signaling pathways. Furthermore, isorhamnetin has been shown to inhibit epithelial–mesenchymal transition, angiogenesis, metastasis, and PI3K/Akt-, MAPK-, STAT3-, and NF-κB-dependent signaling (Figure 6). Importantly, recent studies also emphasize the translational potential of isorhamnetin through nanoformulation-driven delivery systems designed to improve bioavailability, pharmacokinetics, and tumor-targeting efficiency [104].

## 4. Flavonoids as Modulators of p53-Mediated Apoptosis and Cell Cycle Control and Regulators of Bcl-2 Expression

Although numerous studies report flavonoid-mediated modulation of p53 and Bcl-2 family proteins, it is important to recognize that these molecular changes do not necessarily imply direct interaction with the p53–Bcl-2 signaling axis. In many experimental models, flavonoids first induce oxidative stress, mitochondrial dysfunction, DNA damage responses, or metabolic disturbances, which subsequently activate p53-dependent and p53-independent apoptotic pathways. Consequently, altered expression of Bax, Bak, Bcl-2, or Bcl-xL may represent downstream manifestations of broader cellular stress responses rather than primary molecular targets. Therefore, the interpretation of flavonoid-induced apoptosis should consider both direct pathway modulation and indirect stress-mediated mechanisms contributing to apoptotic cell death.

It is estimated that nearly half of all human cancers exhibit mutations or functional inactivation of the p53 tumor suppressor gene, leading to impaired regulation of cell growth, DNA repair, and apoptosis [109,110,111]. Data from the International Agency for Research on Cancer (IARC) database indicate that more than 75% of TP53 mutations are missense variants, with around 97% occurring within the DNA-binding domain (DBD; residues 98–292). Among these, six codons—175, 220, 245, 248, 273, and 282—are identified as major mutation hotspots, each representing over 2% of all recorded missense mutations [112]. In recent years, extensive research has deepened our understanding of the p53 signaling network, revealing its broad influence across diverse cellular processes such as metabolism, immune regulation, and stem cell function. Yet, when mutations occur in p53, they can distort its DNA-binding properties and structural stability, compromising its proper folding and disrupting its normal tumor-suppressive activity [58,113,114]. The p53 has an ability to control the expression of Bcl-2, lowering the concentration of Bcl-2, while increasing the amount of Bax/Bak and leading to apoptosis. For instance, under certain conditions, p53 can function as a repressor of Bcl-2 transcription, resulting in activation of apoptosis [115,116]. The p53 serves as a central regulator of the Bcl-2 family. When activated, whether by DNA damage or compounds like Nutlin-3, it turns on the production of BH3-only pro-apoptotic proteins, such as PUMA (BBC3) and NOXA, which are direct targets of p53. These proteins then block the anti-apoptotic Bcl-2 members, tipping the balance toward mitochondrial membrane permeabilization and triggering caspase-mediated cell death. In pluripotent stem cells, this p53-PUMA/NOXA pathway is particularly active in weaker “loser” cells, making them more prone to apoptosis and lowering their competitive fitness. So, while p53 does not usually control Bcl-2 directly, it drives cell death by switching on its pro-apoptotic partners that neutralize Bcl-2’s protective role [117,118].

### 4.1. Flavonols

Results of the experimental studies investigating the effects of selected flavonols and flavonols-based combinations on p53 signaling, Bcl-2 family proteins, and apoptosis-related outcomes in cancer models can be found in Table 6.

The apparently divergent findings regarding quercetin-mediated regulation of p53 in HepG2 cells should be interpreted in the context of experimental design and the complex, multi-level regulation of p53 signaling. Tanigawa et al. [121] reported increased p53 expression in HepG2 cells, whereas Abdu et al. [124] observed downregulation of p53 gene expression in the same cell line. This discrepancy may result from differences in quercetin concentration, treatment duration, cellular stress intensity, and the experimental endpoint used to assess p53 regulation. Importantly, p53 activity is not determined exclusively by transcriptional expression. In many stress-response models, p53 is primarily regulated at the post-translational level through phosphorylation, acetylation, inhibition of MDM2-mediated degradation, and protein stabilization. Therefore, increased p53 protein accumulation and reduced p53 mRNA expression may occur under different experimental conditions and do not necessarily represent mutually exclusive biological outcomes. Moreover, quercetin can induce apoptosis through mechanisms that are not strictly dependent on p53 transcriptional activation, including ROS generation, mitochondrial membrane potential disruption, modulation of the Bax/Bcl-2 ratio, cytochrome c release, and caspase activation. These observations indicate that quercetin-mediated apoptosis in HCC models is context-dependent and may involve both p53-dependent and p53-independent mechanisms.

### 4.2. Flavones

Results of the experimental studies investigating the effects of selected flavones and flavones-based combinations on p53 signaling, Bcl-2 family proteins, and apoptosis-related outcomes in cancer models can be found in Table 7.

### 4.3. Flavanones

Results of the experimental studies investigating the effects of selected flavanones and flavanones-based combinations on p53 signaling, Bcl-2 family proteins, and apoptosis-related outcomes in cancer models can be found in Table 8.

### 4.4. Anthocyanins

Results of the experimental studies investigating the effects of selected anthocyanins and anthocyanins-based combinations on p53 signaling, Bcl-2 family proteins, and apoptosis-related outcomes in cancer models can be found in Table 9.

### 4.5. Flavanols

Results of the experimental studies investigating the effects of selected flavanols and flavanols-based combinations on p53 signaling, Bcl-2 family proteins, and apoptosis-related outcomes in cancer models can be found in Table 10.

### 4.6. Isoflavones

Results of the experimental studies investigating the effects of selected isoflavones and isoflavones-based combinations on p53 signaling, Bcl-2 family proteins, and apoptosis-related outcomes in cancer models can be found in Table 11.

Comparison of the available evidence (Table 12) suggests that flavonols and flavanols currently provide the strongest support for direct modulation of the p53–Bcl-2 axis, whereas evidence for anthocyanins and certain flavanones remains more limited and frequently involves indirect regulation through oxidative stress and upstream signaling pathways. Across all subclasses, modulation of the Bax/Bcl-2 balance and activation of the intrinsic mitochondrial pathway represent the most consistently reported mechanisms of apoptosis induction.

## 5. Therapeutic Potential and Challenges of Flavonoids in Cancer Treatment

The bioavailability of flavonoids is restricted by their solubility in water. Usually, glucoside forms perform better solubility than aglycones [161]; however, in the case of genistein, the aglycone has higher solubility than the glucoside form [162]. To consider its having a high bioavailability, satisfactory water solubility, low permeability, and stability has to be ensured in case of clinical purposes [163,164]. In vitro effectiveness of flavonoids does not correlate with in vivo results, since it relies on bioavailability [165]. The gut microbiota significantly impacts the bioavailability of polyphenols by enzymatically transforming their chemical forms, including aglycones, glycosides, and conjugated metabolites like O-glucuronides and O-sulfates [166]. Gut microbiota play a critical role in the biotransformation of flavonoids. Consumed food, rich in these compounds, is absorbed only partially in the small intestine [167]. The remaining flavonoids travel to the colon, where gut microbes break them down into simpler phenolic acids such as 3,4-dihydroxybenzoic acid (protocatechuic acid) [168] and 5-(3′-hydroxyphenyl)-γ-valerolactone. These smaller molecules are much easier for the intestinal wall to absorb, meaning that the gut microbiota’s enzymatic activity ultimately determines how much of the flavonoid’s beneficial components the human body can utilize. Interestingly, these microbial metabolites can retain, lose, or even gain new biological activities. In many cases, the original flavonoid is only mildly active, while its microbial breakdown products show stronger antioxidant or anti-inflammatory effects. Some gut-derived compounds, like urolithins formed from ellagitannins, have even been found to inhibit cancer cell migration and protect heart tissue [166].

### 5.1. Modification of Structure

#### 5.1.1. Glycosylation

Changing the structure of the flavonoid by glucosylation results in enhanced water solubility compared to aglycones [169]. Glucosidation, adding a glucose unit to a flavonoid, greatly improves its water solubility because the sugar brings in extra hydroxyl groups that make the molecule more hydrophilic. For example, attaching galactose made myricitrin 480 times more soluble, while adding glucose or maltose increased puerarin’s solubility 14- and 168-fold, respectively. In general, adding glucose boosts solubility, though sugars containing rhamnose can slightly reduce it [170].

#### 5.1.2. Esterification

Transforming the hydroxyl groups of flavonoids into esters, thereby making them more lipid-soluble and better able to associate with cell membranes, serves as a practical way to increase the overall bioavailability of these compounds [171]. A study was conducted, where the researchers created new versions of two flavonoids (6-hydroxyflavanone and 7-hydroxyflavone) by attaching different fatty acids to them and then examined how these changes affected their behavior in cells. They found that adding fatty acids noticeably altered the compounds’ biological activity. Several of the modified flavonoids, especially those linked to oleic acid, show increased cytotoxicity towards prostate cancer (PC3) cells, although they also reduced the viability of healthy keratinocyte (HaCaT) cells. At the same time, the fatty-acid attachments generally weakened the antioxidant properties of the flavonoids, with only one derivative, 6-sorbic flavanone, showing a small improvement [172].

#### 5.1.3. Acylation

Acylation is the process of attaching acyl groups to the hydroxyl groups of a flavonoid molecule. These modifications typically occur at specific positions on the flavonoid structure, including the 3′, 4′, 5′, 3, and 7 hydroxyls on the core rings, as well as the 3″ and 6″ hydroxyls on the sugar units of flavonoid glycosides [173]. Acylation changes flavonoids by adding lipophilic acyl groups, which boost their solubility in lipids and help them cross cell membranes, improving absorption and bioavailability. Acylated sugar parts often enhance antioxidant, anti-inflammatory, and protective effects, and can trigger cancer-cell apoptosis. Modifying the phenolic hydroxyls, however, may reduce antioxidant activity while increasing anti-cancer and anti-inflammatory actions. The impact also depends on the acyl chain: unsaturated or moderately long chains improve membrane activity, while very long saturated chains can lower antioxidant effects [174].

#### 5.1.4. Halogenation

Another modification approach is halogenation, which refers to the chemical modification in which halogen atoms are incorporated into organic molecules like flavonoids, most frequently targeting the 3, 6, 8, 4′, and 3′ positions of the structure [173]. Numerous studies have demonstrated that this process can alter the anticancer and antibacterial properties of flavonoids [164,175]. Introducing electronegative chlorine substantially enhanced antitumor activity. Compared with genistein, chlorination led to a 2.6-fold increase in activity for 8-chlorogenistein (2) and a 7.7-fold increase for 3,8-dichlorogenistein [176]. Another study explored halogenated versions of baicalein and chrysin as potential inhibitors of human protein kinase CK2 (hCK2α). Experiments showed that adding halogen atoms generally improved binding to CK2, with 8-chlorochrysin showing the strongest interaction, even more than the standard inhibitor TBBt. In cell tests, 8-bromobaicalein was most effective against MCF-7 breast cancer cells, while unmodified chrysin worked best against MV4-11 leukemia cells [177]. Chalcones 3 and flavonols 4 with halogen atoms on the B-ring showed stronger activity than similar compounds with methoxy or methyl groups. Their effects were similar to those of the natural flavonoid quercetin, but the halogenated versions were effective at much lower concentrations [178].

### 5.2. Bioavailability Enhancement and Translational Perspectives

Different nanocarrier systems offer distinct advantages for improving flavonoid delivery and pharmacokinetics. Liposomes provide excellent biocompatibility and can enhance the solubility of poorly water-soluble flavonoids, although their long-term stability may be limited. Polymeric nanoparticles generally offer greater structural stability, controlled drug release, and prolonged circulation time. Lipid-based nanoparticles and nanoemulsions can improve intestinal absorption and facilitate systemic delivery of hydrophobic flavonoids. Importantly, many nanocarrier systems increase flavonoid accumulation within tumor tissue through improved pharmacokinetic properties and, in some cases, active targeting strategies involving surface modification with tumor-recognizing ligands. Consequently, nanocarrier-based formulations may substantially improve the therapeutic performance of flavonoids by enhancing bioavailability, reducing premature metabolism, and increasing tumor-specific delivery.

It has been proven that the usage of nanocarriers in the case of delivering quercetin resulted in improvement of overall stability, increased local concentration, and enhanced accumulation in targeted sites, as well as extended circulation time, showing a positive impact on therapeutic efficiency [179,180].

Flavonoids like fisetin have poor water solubility and are rapidly metabolized, resulting in low oral bioavailability. Nanocarrier systems help overcome these issues by improving solubility, protecting fisetin from acidic and enzymatic degradation, and enhancing intestinal absorption. Polymeric nanoparticles (PLA, MPEG-PCL) reduce particle size to the nanometer range, increasing surface area and dissolution rate, which extends fisetin’s half-life and boosts tumor accumulation in vivo. Polymeric micelles made from amphiphilic copolymers encapsulate fisetin with over 98% efficiency, producing 1.8- to 6.3-fold increases in C_max, t_1/2, and AUC, along with stronger anticancer activity [181,182]. Self-nanoemulsifying drug delivery systems (SNEDDS) form fine oil-in-water droplets during digestion, improving oral absorption by up to 3.7-fold. Lipid-based carriers like liposomes and solid lipid nanoparticles further enhance lymphatic transport and protection from enzymatic breakdown, achieving up to an eight-fold increase in AUC [181,183,184]. Cyclodextrin inclusion complexes use their hydrophobic cavities to solubilize fisetin, raising solubility to 162-fold, while nanocrystals expand surface area, increasing solubility from 60 µg/mL to over 400 µg/mL and accelerating dissolution [181,185].

In a study examining nanoformulated chrysin, chrysin-loaded PLGA-PVA nanoparticles were successfully produced and shown to be cytocompatible while retaining strong antioxidant and DNA-protective effects. The nanochrysin also displayed significantly greater inhibition of breast and ovarian cancer cells than free chrysin by inducing apoptosis. With its high encapsulation efficiency, small particle size, and sustained-release behavior, this formulation shows promise as a potential cancer drug-delivery system [186].

Unwanted off-topic effects of flavonoids, besides proapoptotic use in cancer treatment, can occur during systemic use, such as chelating iron by catechins, which can lead to iron deficiency [187]. The application of nanocarriers not only enhances the bioavailability of therapeutic compounds but also minimizes the potential for adverse effects.

Despite promising anticancer activity, the clinical translation of flavonoids remains limited due to poor aqueous solubility, rapid metabolism, low chemical stability, short systemic half-life, and insufficient tumor-selective accumulation. Recent advances in nanotechnology-based delivery systems have therefore attracted considerable attention as a strategy to overcome these pharmacokinetic limitations while preserving the biological activity of flavonoids. Polymeric nanoparticles, liposomes, solid lipid nanoparticles, nanoemulsions, micelles, and hybrid nanocarriers have been shown to substantially improve flavonoid stability, bioavailability, cellular uptake, circulation time, and targeted tumor delivery. In addition, surface-functionalized nanoparticles may enhance tissue selectivity and intracellular accumulation, particularly in difficult-to-treat tumors and multidrug-resistant cancer models.

Importantly, nanoformulation approaches do not merely improve pharmacokinetics but may also enhance apoptosis-related anticancer mechanisms. Nanoencapsulated flavonoids have demonstrated increased ability to induce mitochondrial dysfunction, ROS accumulation, cytochrome c release, Bax/Bcl-2 modulation, caspase-3/-9 activation, and inhibition of pro-survival signaling pathways including PI3K/Akt, NF-κB, MAPK, and STAT3. Recent studies involving isorhamnetin nanoformulations further suggest improved regulation of angiogenesis, epithelial–mesenchymal transition, and metastatic signaling while simultaneously enhancing intracellular delivery efficiency. Similarly, flavonoid-loaded nanoparticles designed for glioblastoma therapy have shown improved penetration across biological barriers and enhanced pro-apoptotic activity compared with free flavonoids. These findings indicate that nanoformulation strategies may substantially improve the translational potential of flavonoid-based apoptosis-targeting therapies in oncology.

Different nanoformulation strategies vary considerably in their ability to improve flavonoid pharmacokinetics and biological activity. Liposomes offer excellent biocompatibility and can enhance solubility and systemic circulation; however, their long-term stability may be limited. Polymeric nanoparticles generally provide superior structural stability, controlled release, and protection against premature metabolic degradation. Nanoemulsions and micellar systems may improve intestinal absorption and facilitate intracellular delivery of hydrophobic flavonoids, although their drug-loading capacity can be more limited. Lipid-based nanoparticles combine favorable biocompatibility with efficient encapsulation and enhanced tumor accumulation. By increasing intracellular flavonoid concentrations, these delivery systems may strengthen modulation of apoptosis-related pathways, including p53 activation, regulation of Bax/Bcl-2 family proteins, mitochondrial dysfunction, and caspase activation. Based on currently available evidence, polymeric nanoparticles and lipid-based nanocarriers appear to offer the greatest translational potential due to their ability to simultaneously improve stability, pharmacokinetics, tumor targeting, and therapeutic efficacy.

Despite their promising biological activities, the clinical application of flavonoids is frequently limited by unfavorable pharmacokinetic properties. Following oral administration, many flavonoids exhibit poor intestinal absorption and extensive presystemic metabolism. In addition, gut microbiota play a significant role in flavonoid biotransformation, generating metabolites that may differ substantially from the parent compounds in terms of biological activity. After absorption, flavonoids commonly undergo phase II metabolic reactions, including glucuronidation, sulfation, and methylation, which further modify their pharmacological properties and reduce the concentration of free bioactive molecules in circulation. Rapid systemic clearance and extensive metabolism often result in low plasma concentrations despite relatively high dietary intake. Consequently, bioavailability remains a major challenge that must be addressed to fully exploit the therapeutic potential of flavonoids in cancer treatment.

### 5.3. Flavonoids in Combination Therapy and Apoptosis Sensitization

Increasing evidence suggests that flavonoids may enhance the efficacy of conventional anticancer therapies when used in combination with chemotherapeutic agents, radiotherapy, or targeted therapies. The ability of flavonoids to modulate apoptosis-related signaling pathways makes them particularly attractive as chemosensitizing agents capable of overcoming therapy resistance and improving tumor cell susceptibility to treatment-induced apoptosis.

Several flavonoids, including quercetin, kaempferol, myricetin, and isorhamnetin, have demonstrated synergistic interactions with standard anticancer agents through mechanisms involving p53 stabilization, suppression of anti-apoptotic Bcl-2 family proteins, increased Bax/Bcl-2 ratio, mitochondrial membrane depolarization, ROS accumulation, cytochrome c release, and activation of caspases-3, -8, and -9. In addition, flavonoids may inhibit pro-survival signaling pathways such as PI3K/Akt/mTOR, NF-κB, MAPK, and STAT3, thereby sensitizing cancer cells to chemotherapy-induced stress and reducing resistance mechanisms.

Recent studies further suggest that flavonoids may improve the therapeutic response to targeted therapies and immune-related treatments by modulating oxidative stress, inflammatory signaling, epithelial–mesenchymal transition, and tumor microenvironment dynamics. Importantly, nanoformulation-based delivery systems may additionally enhance combination efficacy by improving intracellular flavonoid accumulation and tumor-selective delivery. Although most evidence remains preclinical, these findings indicate that flavonoid-based combination strategies may represent a promising approach for improving apoptosis-targeted cancer therapy while potentially reducing systemic toxicity associated with high-dose conventional treatment.

### 5.4. Clinical Evidence and Translational Challenges

Despite extensive preclinical evidence demonstrating the anticancer potential of flavonoids, clinical validation remains limited. Most available human studies have focused on dietary supplementation, safety assessment, pharmacokinetics, or biomarker evaluation rather than direct analysis of apoptosis-related mechanisms in tumors. Several flavonoids, including quercetin, genistein, and epigallocatechin gallate (EGCG), have been investigated in clinical settings, particularly in prostate, breast, colorectal, and other solid tumors. However, the available studies are generally characterized by small patient cohorts, heterogeneous study designs, variable dosing regimens, and limited mechanistic endpoints.

Importantly, no clinical study has conclusively demonstrated direct modulation of the p53–Bcl-2 signaling axis in cancer patients following flavonoid administration. Therefore, although preclinical findings strongly support the ability of flavonoids to regulate apoptosis-related pathways, a substantial gap remains between experimental evidence and clinical applicability. Future studies should focus on biomarker-guided clinical trials, optimization of bioavailability, molecular stratification of patients according to p53 status, and evaluation of flavonoids as components of combination therapies [188,189,190].

## 6. Conclusions and Future Perspectives

The evidence reviewed in this work indicates that flavonoids are capable of modulating apoptosis in cancer cells through multiple interconnected mechanisms centered around the p53–Bcl-2 signaling axis. Although individual compounds differ in potency and molecular targets, the most consistently reported effects include stabilization or activation of p53, increased expression of pro-apoptotic proteins such as Bax and Bak, suppression of anti-apoptotic proteins including Bcl-2, Bcl-xL, and Mcl-1, mitochondrial outer membrane permeabilization, cytochrome c release, and activation of caspase-dependent apoptotic pathways. These effects are frequently accompanied by modulation of oxidative stress and inhibition of pro-survival signaling networks such as NF-κB, PI3K/Akt, and STAT3. Among the flavonoid subclasses discussed, flavonols (particularly quercetin and kaempferol) and flavanols (especially EGCG) currently provide the strongest evidence for direct modulation of apoptosis-related pathways associated with the p53–Bcl-2 network. However, growing evidence suggests that flavonoid-induced apoptosis is not restricted to p53-dependent mechanisms and may also occur in p53-mutant or p53-deficient cancers through ROS-mediated mitochondrial dysfunction, direct regulation of Bcl-2 family proteins, and suppression of survival signaling pathways. Despite extensive preclinical evidence, significant translational challenges remain. Poor bioavailability, rapid metabolism, limited tumor-specific delivery, and the scarcity of robust clinical data continue to hinder therapeutic application. Future research should focus on biomarker-guided studies, evaluation of flavonoid activity in molecularly defined cancer subtypes, particularly those characterized by TP53 alterations, development of advanced delivery systems, and well-designed clinical trials capable of validating the promising mechanistic findings observed in experimental models. Such approaches may facilitate the translation of flavonoid-based strategies from experimental research to clinically relevant anticancer therapies.

## Figures and Tables

**Table 1:** Classification of Bcl-2 family proteins.

Bcl-2		
Proapoptotic		Antiapoptotic
BH3-only	BH123	
BID, BIM, BAD, BIK, PUMA, NOXA	BAX, BAK	BCL-xL, MCL1, BCL-W, BCL-B, BCL2A1

**Table 2:** Comparison between the intrinsic and extrinsic pathways of apoptosis.

Feature	Intrinsic Pathway (Mitochondrial Pathway)	Extrinsic Pathway (Death Receptor Pathway)	Source
Main Trigger	Internal cellular stress (DNA damage, oxidative stress, growth factor deprivation, ER stress)	External signals (binding of death ligands to cell-surface death receptors)	[28,40]
Primary Stimuli	Radiation, toxins, hypoxia, oncogene activation, severe DNA damage	Fas ligand (FasL), Tumor Necrosis Factor (TNF), TRAIL (TNF-related apoptosis-inducing ligand)	
Key Initiator Molecules	Bcl-2 family proteins (pro-apoptotic: Bax, Bak; anti-apoptotic: Bcl-2, Bcl-xL)	Death receptors (Fas/CD95, TNFR1, DR4, DR5) and adaptor proteins (FADD, TRADD)	[30,41]
Critical Events	Mitochondrial outer membrane permeabilization (MOMP), release of cytochrome c into the cytosol	Formation of the death-inducing signaling complex (DISC) after ligand binding	[28,35]
Initiator Caspase	Caspase-9	Caspase-8	[34,35]
Executioner Caspases	Caspase-3, Caspase-6, Caspase-7		[32]
Regulation/Inhibitors	Bcl-2	FLIP proteins can inhibit DISC formation	[29]
Energy Dependence	ATP-dependent		[43]
Morphological Features	Cell shrinkage, chromatin condensation, membrane blebbing, apoptotic bodies		[28]

**Table 3:** TP53 status of representative cancer cell models discussed in this review and their relevance for interpretation of flavonoid-induced apoptotic responses. Abbreviations: WT, wild-type TP53; Mut, mutant TP53; Null, TP53-deficient/null.

Frequently Used Cancer Cell Line	TP53 Status
HepG2	WT
Huh7	Mut
MCF-7	WT
MDA-MB-231	Mut
PC3	Null
H1299	Null
A549	WT

**Table 4:** Mechanistic structure–activity relationships of flavonoids relevant to modulation of the p53–Bcl-2 axis and apoptosis induction in cancer cells.

Structural Feature	Biological Consequence
Catechol moiety (3′,4′-OH)	ROS modulation, p53 activation
C2=C3 + 4-oxo	Stronger apoptotic signaling
Methoxylation	Increased lipophilicity, uptake
Glycosylation	Lower uptake, altered bioavailability
Gallate group	Enhanced Bcl-2 modulation, caspase activation

**Table 5:** Structure of flavonoids.

Flavonoid	Structure
Flavones	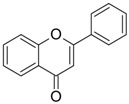
Flavonols	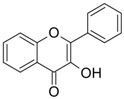
Flavanones	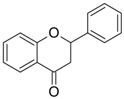
Isoflavones	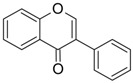
Anthocyanins	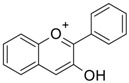
Flavanols	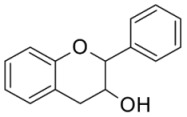
Chalcones	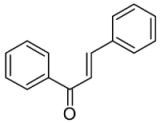
Kaempferol	** 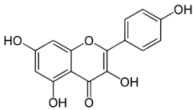 **
Epigallocatechin gallate (EGCG)	** 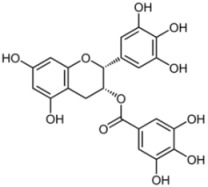 **
Quercetin	** 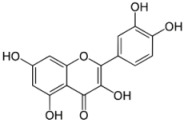 **

**Table 6:** Summary of experimental studies investigating the effects of selected flavonols and flavonols-based combinations on p53 signaling, Bcl-2 family proteins, and apoptosis-related outcomes in cancer models.

Compound	Experimental Model (Cells/Organism)	Effect on p53	Effect on Bcl-2/Apoptotic Pathway	Biological Outcome (Cell Cycle/Apoptosis)	Source
Quercetin + venetoclax	AML cell lines: KG-1, Kasumi-1	Not directly assessed.	AML cells show high Bcl-2 expression (TCGA, MILE, DepMap). Quercetin ↑ Bax, slightly ↓ Bcl-2, no effect on Bak, Survivin, or other IAPs. Combination enhances Bax/Bcl-2 ratio, sensitizing cells to Venetoclax-induced apoptosis.	Combination treatment caused strong sub-G_1_ accumulation (>70%), decreased G_1_ phase, and marked nuclear condensation; Quercetin alone had a mild effect, but synergistically enhanced Venetoclax-induced cell death.	[119]
Quercetin	KON oral cancer cells (human)	Not directly assessed.	↓ BCL-2 and BCL-XL expression; ↑ BAX expression, increasing the BAX/BCL-2 ratio; Annexin V-positive cells rise, indicating apoptosis	Treatment resulted in a marked increase in reactive oxygen species (ROS) generation, as detected by DCFDA staining and flow cytometry analysis. This was accompanied by a loss of mitochondrial membrane potential, indicated by reduced Rhodamine 123 uptake. Flow cytometric assessment of DNA content revealed cell-cycle arrest at the S and G_2_/M phases, consistent with disruption of cell-cycle progression.	[120]
Quercetin	Human hepatoma HepG2 cells (wild-type p53)	↑ total p53 protein and Ser15 phosphorylation; half-life extended from 74 min to 184 min; ubiquitination markedly reduced → p53 stabilization	↑ Bax, ↓ Bcl-2 protein and phospho-Bcl-2, raising Bax/Bcl-2 ratio; caspase-3 cleavage observed	G_2_/M arrest (↑ G_2_/M, ↓ S phase) and DNA fragmentation (36–48 h), indicating apoptosis	[121]
Quercetin	MCF-7 breast cancer cells	Not directly assessed.	Bcl-2 down-regulated; pro-apoptotic Bax and Caspase-3 up-regulated	↑ Apoptosis with nuclear condensation, chromatin fragmentation, and increased Annexin-V-positive cells; suppression of EGFR/PI3K/Akt signaling leads to reduced proliferation	[122]
Quercetin	MDA-MB-231 breast cancer cells	Not directly assessed.	Same pattern as MCF-7: Bcl-2 down-regulated; Bax and Caspase-3 up-regulated	↑ Apoptosis with similar morphological changes; EGFR/PI3K/Akt inhibition also decreases Cyclin D1 via GSK-3β activation, suggesting cell-cycle arrest mechanisms	
Quercetin	Human cervical cancer (HeLa) cells	Upregulation of p53 expression; activation of p53–p21 pathway	↓ Bcl-2, ↓ Bcl-xL, ↓ Mcl-1, ↓ p-Bad, ↓ survivin; ↑ Bax, ↑ Bad, ↑ Apaf-1, ↑ cytosolic cytochrome c, ↑ caspase-9, ↑ caspase-3, ↑ PARP cleavage	G_2_/M phase cell cycle arrest via p53–p21 activation; Mitochondria-mediated apoptosis (intrinsic pathway) through cytochrome c release and caspase activation	[123]
Quercetin	HepG2 human HCC cells (in vitro); DEN/2-AAF-induced HCC in rats (in vivo)	Downregulated p53 gene expression in vivo (restored to near-normal levels); the reduction was attributed to cell recovery rather than direct p53 activation	Induced late apoptosis and necrosis; caspase activation, regulation of Bcl-2, and inhibition of PI3K/Akt and ERK pathways	Arrested cell cycle at G_1_ and S phases; IC50 = 107.7 µM; improved liver enzymes and lipid profile; decreased VEGF and NF- κB	[124]
Quercetin + Sorafenib		Downregulated p53 gene expression (along with VEGF and NF-κB); more effective than sorafenib alone	Highest apoptosis and necrosis rate; synergistic effect	Arrested cell cycle at S phase; IC50 = 9.98 µM; best restoration of liver structure, enzymes, and lipid profile; reduced inflammation	
Kaempferol	SCC-9 (tongue squamous-cell carcinoma)	Not directly assessed.	↓ Bcl-2 protein expression; ↑ cytochrome c release; ↑ active caspase-3 (3.3% → 31.4% at IC_50_	Kaempferol induces S-phase arrest (G_1_ ↓, S ↑); Fisetin shifts cells to G_2_/M and sub-G_1_ phases	[125]
Kaempferol	Recombinant human BAX and Bcl-2 proteins immobilised on AFM tips and Au substrates (single-molecule force spectroscopy)	Not directly assessed.	Kaempferol reduces the BAX/Bcl-2 binding probability and lowers the specific unbinding force from ~71 pN (control) to 20.72 pN, indicating a ∼35% decrease in binding strength; the overall binding affinity is weakened (≈10-fold reduction in BP for polyphenols, with kaempferol showing a modest effect)	The disruption of the BAX/Bcl-2 interaction is interpreted as a release of the anti-apoptotic brake, suggesting increased susceptibility to mitochondrial-mediated apoptosis (no direct cell-based apoptosis assay performed)	[126]
Kaempferol	MC-3 human oral-cancer cells (in vitro)	Not directly assessed.	↓ Bcl-2 protein, ↑ Bax protein, resulting in an increased Bax/Bcl-2 ratio (indicative of intrinsic apoptosis)	Dose-dependent reduction in cell viability, ↑ apoptotic bodies (DAPI) and annexin V-positive cells, indicating activation of intrinsic apoptosis	[127]
	BALB/c nude mice bearing MC-3 xenografts (in vivo)	Not directly assessed.	Tumor tissues showed ↓ Bcl-2 and ↑ pro-apoptotic markers (consistent with in vitro findings)	Significant decrease in tumor volume without liver or kidney toxicity, increased TUNEL-positive apoptotic cells.	
Kaempferol	HeLa cells (Human cervical cancer)	Upregulated p53 expression (at both transcript and protein levels); phosphorylation at Ser15 increased.	Downregulated anti-apoptotic Bcl-2, XIAP, Livin, cIAP-2; Upregulated pro-apoptotic Bad, Bax, Bid, Bim, Cyt-c, Caspase-3, Caspase-8, Caspase-9, APAF1; Reduced mitochondrial membrane potential	Induced G_2_/M cell cycle arrest; increased early apoptosis (up to 25% at 50 µM, 48 h); DNA fragmentation and nuclear blebbing observed; activation of both intrinsic and extrinsic apoptotic pathways.	[128]
Kaempferol	HepG2 cells treated with kaempferol-coated AgNPs (≈200 nm)	p53 protein level markedly increased (significant elevation)	Bcl-2 expression significantly reduced; pro-apoptotic markers Bax, cytochrome-c, and caspase-3 markedly elevated, indicating activation of the intrinsic apoptotic cascade	Oxidative-stress-mediated apoptosis with accompanying cell-cycle arrest; LDH leakage and ROS/LPO rise confirm cytotoxicity	[129,130]
Myricetin	OVCAR-3 (cisplatin-resistant ovarian cancer)	↑ p53 protein (and p21) → p53-dependent apoptosis	↓ Bcl-2 and Bcl-xl; ↑ Bax and Bad; ↑ DR5 ↓ procaspase-8 → activation of intrinsic (Bcl-2 family) and DR5-mediated extrinsic pathways	Strong apoptosis (≈84% at 30 µM); no cell-cycle arrest	[131]
	A2780/CP70 (cisplatin-resistant ovarian cancer)	↑ p53 protein	↓ Bcl-2 and Bcl-xl; ↑ Bax and Bad; intrinsic pathway activated; no DR5 or procaspase-8 change	Apoptosis (≈42% at 30 µM); no cell-cycle arrest	
	IOSE-364 (normal ovarian epithelial)	No detectable p53 change (myricetin spared normal cells	No significant Bcl-2 family alteration; apoptosis not induced	Viability largely unchanged; no apoptosis	
Fisetin	SCC-25 (tongue squamous-cell carcinoma)	Not directly assessed.	↓ Bcl-2 signal; ↑ cytochrome c (modest); ↑ active caspase-3 (10.2% at IC_50_)	Fisetin causes G_2_/M arrest and sub-G_1_ increase; Kaempferol reduces G_1_ population	[125]
Galangin (free drug)	AGS and BGC823 human gastric cancer cells	Not directly reported for free galangin alone	Bax, Bcl-2, and p53 proteins were detected by western blot after galangin treatment	Induces apoptosis, inhibits clonogenesis, migration, and invasion at 50 µM	[132]
Galangin (exosome-encapsulated)	AGS and BGC823 human gastric cancer cells (in vitro); female nude mice with BGC823 xenograft (in vivo)	Up-regulates p53 expression (mRNA and protein) by down-regulating miR-10b-5p, which has a binding site with the p53 gene	Bax, Bcl-2, and p53 proteins were detected by western blot; apoptosis mediated via miR-10b-5p/p53 axis	Promotes apoptosis, suppresses proliferation and invasion of AGS and BGC823 cells; in vivo, 500 µM/mouse galangin-exosome significantly reduced tumor volume with no pathological changes to major organs	

**Table 7:** Summary of experimental studies investigating the effects of selected flavones and flavones-based combinations on p53 signaling, Bcl-2 family proteins, and apoptosis-related outcomes in cancer models.

Compound	Experimental Model (Cells/Organism)	Effect on p53	Effect on Bcl-2/Apoptotic Pathway	Biological Outcome (Cell Cycle/Apoptosis)	Source
Nobiletin	MCF-7 human breast-cancer cells (in vitro)	p53 protein expression was significantly up-regulated after nobiletin treatment	Bcl-2 expression decreased, while Bax and cleaved caspase-3 levels increased, indicating activation of the intrinsic apoptotic cascade	Dose-dependent reduction in cell viability and a marked rise in apoptosis together with inhibition of migration	[133,134]
5,3 0 -dihydroxy-3,6,7,8,4 0 -pentamethoxyflavone (PMF)	MCF-7 breast cancer cells (human)	↑ p53 protein levels (significant at 20 µM)	↓ BCL-2 expression; ↑ BAX and cytochrome c; activation of caspase-3/-7 and PARP-1 cleavage → intrinsic apoptosis	G_1_-phase cell-cycle arrest (↑ 14.9% in G_1_) and robust induction of apoptosis (loss of mitochondrial membrane potential, ROS over-production)	[135]
Apigenin	K562 chronic myeloid leukemia cells; FS-2 normal human B lymphoblastoid cells (in vitro)	P53 mRNA significantly upregulated compared to control	↑ Bax, ↓ Bcl-2 (2–4 fold); increased BAX/BCL-2 ratio; induced caspase-3, 6, 7, 9 expression	Dose-dependent reduction in cell viability (up to 85%); IC50 = 86.29 µM at 24 h; apoptotic morphology (cell shrinkage, chromatin fragmentation, crescent nuclei); no significant toxicity to normal FS-2 cells	[136]
Apigenin + doxorubicin			BAX expression lower than DOX alone (1.6–1.8 vs. 2.56-fold); BCL-2 downregulated; BAX/BCL-2 ratio lower than single treatments; caspase-3, 6, 7, 9 expression lower than DOX alone; however, cleaved PARP-1 and cleaved caspase-3 detected by western blot	~60% reduction in cell viability; synergistic (CI = 0.92–0.97); more growth inhibition than DOX alone; apoptotic morphology preserved; no significant toxicity to normal FS-2 cells	
Apigenin	HepG-2 human hepatocellular carcinoma cells; HeLa cervical cancer cells (in vitro)	Significantly increased p53 gene expression	Bcl-2 protein decreased in a concentration-dependent manner; caspase-9 increased; p53/Bcl-2/caspase-9 apoptotic pathway confirmed	G_2_/M phase arrest with increased pre-G_1_ (apoptotic) phase; IC50 = 57.86 µg/mL (HepG-2); increased SOD 1.84-fold; CAT inhibited 27.13%; induced apoptosis	[137]
Encapsulated apigenin nanoparticles—chitosan NPs coated with folic acid-conjugated BSA		Significantly higher p53 gene expression than free apigenin	Bcl-2 decreased in a concentration-dependent manner (confirmed by IHC); caspase-9 significantly overexpressed (higher than free Ap); p53/Bcl-2/caspase-9 pathway established; MMP9 downregulated (anti-metastatic)	S phase arrest (different from free Ap’s G_2_/M arrest); IC50 = 11.49 µg/mL (5-fold more potent than free Ap); higher apoptosis rate than free Ap (mostly apoptosis, not necrosis); SOD increased 2.4-fold; CAT inhibited 59.36%; synergistic with DOX (IC50 7.76 µg/mL + DOX); targeted delivery via folate receptor	
Apigenin	Human DLBCL OCI-LY3 cells (in vitro); BALB/c mouse xenograft model (in vivo)	Not investigated in this study	Bax ↑ (40, 80 µmol/L) Bcl-2 ↓ (40, 80 µmol/L) Cleaved caspase-3 ↑ (40, 80 µmol/L) → Intrinsic mitochondrial pathway activated	In vitro: Concentration- and time-dependent proliferation inhibition (MTT), Dose-dependent migration and invasion suppression (Transwell), Apoptosis rate ↑, highest at 80 µmol/L (flow cytometry). In vivo: Reduced xenograft tumor volume and weight; Ki-67 ↓ in tumor tissue	[138]
Vitexin	NB-4 (AML) and MOLT-4 (ALL) leukemic cell lines; primary bone marrow cells from AML and ALL patients (in vitro)	Not investigated in this study	HIF-1α ↓ (mRNA and protein) Bcl-2 ↓ (mRNA) Caspase-3 mRNA ↑ Pro-caspase-3 ↓, Cleaved caspase-3 ↑ (protein) → HIF-1α/Bcl-2/caspase-3 pathway modulated	Cell viability ↓ dose- and time-dependently (MTT) IC_50_ (48 h): 901 µM (NB-4), 929 µM (MOLT-4). Minimal toxicity to normal PBMCs. Apoptosis: 42.82% (NB-4), 40.0% (MOLT-4). Synergistic with daunorubicin in NB-4 (CI = 0.8); additive in MOLT-4 (CI = 1.0). In patient cells: 19.88% (AML), 17.73% (ALL) apoptosis; combination ↑ to 22.15% (AML), 18.82% (ALL)	[139]

**Table 8:** Summary of experimental studies investigating the effects of selected flavanones and flavanones-based combinations on p53 signaling, Bcl-2 family proteins, and apoptosis-related outcomes in cancer models.

Compound	Experimental Model (Cells/Organism)	Effect on p53	Effect on Bcl-2/Apoptotic Pathway	Biological Outcome (Cell Cycle/Apoptosis)	Source
Hesperidin	Human pre-B NALM-6 cells (wild-type p53)	Hesperidin ↑ p53 mRNA and protein levels	↑ Bax, ↓ Bcl-2, ↓ XIAP; dose-dependent cleavage of procaspase-3 and procaspase-9, indicating activation of the intrinsic caspase cascade	↓ cell viability and proliferation; accumulation of cells in sub-G_0_/G_1_ (apoptotic) fraction and cell-cycle arrest, reflecting growth inhibition and apoptosis	[140,141]
Liquiritigenin	Human cervical carcinoma (HeLa) cells treated with liquiritigenin	p53 protein expression is markedly increased after 48 h of treatment.	Bax is up-regulated, Bcl-2 is down-regulated, raising the Bax/Bcl-2 ratio. Cytochrome c is released from mitochondria to the cytosol. Caspase-9 and caspase-3 are cleaved/activated, leading to PARP cleavage.	Induction of apoptosis evidenced by chromatin condensation, nuclear fragmentation, Annexin-V/PI positivity, and a dose-dependent rise in apoptotic cells (12–46% vs. 5.8% control)	[142]

**Table 9:** Summary of experimental studies investigating the effects of selected anthocyanins and anthocyanins-based combinations on p53 signaling, Bcl-2 family proteins, and apoptosis-related outcomes in cancer models.

Compound	Experimental Model (Cells/Organism)	Effect on p53	Effect on Bcl-2/Apoptotic Pathway	Biological Outcome (Cell Cycle/Apoptosis)	Source
Anthocyanins from Vaccinum meridionale	Human colon adenocarcinoma SW480 cells	p53 protein is up-regulated and phosphorylated at Ser15 (significant vs. control)	Total BAD protein increased (~1.6–2-fold) while phosphorylated BAD (Ser112) is inhibited, favouring pro-apoptotic signaling	Dose-dependent antiproliferative effect (IC_50_ = 8% *v*/*v*) with time-dependent loss of viability, caspase-3 activation, PARP cleavage, ↑ ROS and ↓ GSH/GSSG ratio → apoptosis	[143]
Dracorhodin perchlorate	Human melanoma A375-S2 cells	Accumulation of p53 protein, accompanied by increased Ser-15 phosphorylation	↑ Bax/↓ Bcl-2 (higher Bax/Bcl-2 ratio); activation of caspase-3 and -8, degradation of ICAD-L and PARP; sustained phosphorylation of JNK and p38 MAPKs (ERK unchanged)	Morphological hallmarks of apoptosis (membrane blebbing, nuclear condensation), DNA fragmentation and loss of viability; p21^WAF1 up-regulated (cell-cycle arrest component)	[144]
Anthocyanins from Pomegranate Seeds and Peel (ethanolic extracts)	HepG2 liver cancer cells (Pomegranate seed ethanolic extract)	↑ p53 mRNA and protein expression (significant up-regulation)	↓ Bcl-2 (anti-apoptotic) and ↑ pro-apoptotic Bax, Casp-3, cytochrome-c (significant up-regulation)	G_0_/G_1_ and S-phase arrest; marked increase in early and late apoptosis	[145]
	HepG2 liver cancer cells (Pomegranate peel ethanolic extract)	↑ p53 mRNA and protein expression (significant up-regulation)	↓ Bcl-2 (anti-apoptotic) and ↑ pro-apoptotic Bax, Casp-3, cytochrome-c (significant up-regulation)	G_0_/G_1_ and S-phase arrest (less pronounced than seed); increase in apoptosis	
Anthocyanins extracted from black soybean	DU-145 prostate cancer cells (in vitro)	↑ p53 expression after anthocyanin treatment	↓ Bcl-2 protein, ↑ Bax protein, resulting in a higher Bax/Bcl-2 ratio	Dose-dependent induction of apoptosis (DNA laddering) and growth inhibition (MTT IC_50_ ≈ 60–90 µM)	[146]
	DU-145 xenograft in athymic nude mice (in vivo)	↑ p53 observed in tumor tissue (consistent with in vitro)	Same pattern of ↓ Bcl-2, ↑ Bax, elevated Bax/Bcl-2 ratio	Significant reduction of tumor volume (≈65% inhibition at 12 weeks), indicating apoptosis-mediated tumor growth suppression	

**Table 10:** Summary of experimental studies investigating the effects of selected flavanols and flavanols-based combinations on p53 signaling, Bcl-2 family proteins, and apoptosis-related outcomes in cancer models.

Compound	Experimental Model (Cells/Organism)	Effect on p53	Effect on Bcl-2/Apoptotic Pathway	Biological Outcome (Cell Cycle/Apoptosis)	Source
Flavanols from Camellia sinensis	MCF-7 (wild-type p53)	↑ p53 protein expression after 24 h GTE (42% EGCG, 40% EGC) treatment	Not directly measured in this study; p53 activation is known to promote pro-apoptotic signaling (Bax/Bcl-2 shift) in other models	↓ cell viability (IC_50_ ≈ 324 µg mL^−1^, 24 h) 3; ↑ p21 → G_1_-phase arrest; reduced migration (≈30% inhibition)	[147]
	MDA-MB-231 (mutant p53-p.R280K)	Redistribution of mutant p53 from nucleus; overall ↓ p53 staining after GTE 1	Not assessed; mutant p53 loss does not alter p21 levels, suggesting limited apoptotic activation	↓ cell viability (IC_50_ ≈ 133 µg mL^−1^, 24 h) 3; strong migration inhibition (≈50% reduction)	
	MCF-10A (non-tumoral)	No significant change in p53 expression (GTE selective for tumor cells)	Not evaluated; cell viability unchanged, indicating lack of cytotoxic/apoptotic effect	No cytotoxicity; viability comparable to control 7	
Epigallocatechin gallate (EGCG)	Purified full-length p53 and N-terminal domain (in vitro SPR/NMR)	EGCG binds the intrinsically disordered NTD with KD ≈1.6 µM and blocks the p53-MDM2 interface, inhibiting MDM2-mediated ubiquitination and stabilising p53	Stabilised p53 can transactivate pro-apoptotic genes (Bax) and antagonise anti-apoptotic Bcl-2 signalling (p53 is known to bind Bcl-2 family proteins)	p53 accumulation leads to transcription of cell-cycle arrest and apoptosis genes; in vitro assays predict enhanced apoptotic response	[148]
	Human lung cancer cells (reported literature)	EGCG disrupts the p53-MDM2 interaction, reducing p53 degradation in cells	p53 activation downstream of EGCG promotes apoptosis by modulating the Bcl-2 family (up-regulating Bax, down-regulating Bcl-2)	Induction of apoptosis and inhibition of proliferation in lung cancer cells	
	In-vitro ubiquitination assay (full-length p53 + MDM2)	EGCG inhibits MDM2-catalysed p53 ubiquitination with an IC_50_ ≈ 100 µM	By preventing ubiquitination, p53 remains active to trigger the apoptotic cascade, including Bcl-2 family regulation	Dose-dependent suppression of p53 ubiquitination leads to increased apoptosis	
	Cell-free SPR competition (MDM2 immobilised, p53 pre-incubated with EGCG)	EGCG pre-binding to p53 reduces p53-MDM2 binding (IC_50_ ≈ 0.5 µM)	Loss of p53-MDM2 interaction frees p53 to activate apoptotic transcription programs	Strong inhibition of the p53-MDM2 interaction predicts cell-cycle arrest and apoptotic induction	
Epigallocatechin-3-gallate	SKOV3/DDP cells (cis-platin-resistant)—EGCG treatment	↑ p53 expression	↑ apoptosis (2.3-fold increase)—Bcl-2 not reported	Inhibited proliferation, migration and invasion; increased apoptotic rate	[149]
	A2780/DDP cells (cis-platin-resistant)—EGCG treatment	↑ p53 expression	↑ apoptosis (2.1-fold increase)—Bcl-2 not reported	Suppressed cell growth, migration and invasion; higher apoptosis	
	SKOV3/DDP and A2780/DDP—S100A4 over-expression + EGCG	↓ p53 (reversal of EGCG-induced increase)	↓ apoptosis (reduced apoptosis ratio)—Bcl-2 not reported	Restores proliferative and migratory abilities, counteracting EGCG effects	
	A2780/DDP xenograft mice—EGCG treatment	↑ p53 in tumor tissue	↑ apoptosis (implied by tumor shrinkage)—Bcl-2 not reported	↓ tumor volume and weight, reduced KI-67 and S100A4/NF-κB, overall tumor growth inhibition	
Epigallocatechin-3-gallate	Human pancreatic cancer cell lines MIAPaCa-2 and SU 86.86; 10–100 µM for 24–48 h	Not assessed in this study	Repressed BCL-2 mRNA expression (an NF-κB target gene) along with MMP9, MMP2, and cMyc, measured by qPCR. Increased apoptosis via NF-κB inhibition.	Apoptosis: Concentration-dependent increase—MIAPaCa-2: 3.13% (20 µM) to 34.85% (100 µM); SU 86.86: 4.92% (20 µM) to 49.05% (100 µM) after 24 h. Cell growth: Significant reduction in viability and proliferation; IC50 = 73 µM (MIAPaCa-2) and 59 µM (SU 86.86). Cell cycle inhibition also observed	[150]
Epigallocatechin-3-gallate	C57BL/6 female mice with 4-NQO-induced oral carcinogenesis; 686Tu (malignant SCCHN), MSK (premalignant) cells	Minimal effect on gene expression; p53 pathway not significantly activated by EGCG alone	Minimal effect on apoptosis-related gene expression	Did not significantly reduce visible or microscopic lesions at 30 mg/kg	[151]
EGCG + resveratrol		p53 pathway significantly upregulated	Apoptosis pathway among the top enriched hallmarks	Significantly inhibited both visible lesions (0.8/mouse) and microscopic lesion number and area	
(-)-Epicatechin (EC)	4T1 murine triple-negative breast cancer cells (in vitro); C2C12 mouse myoblasts used as non-tumor control	Not reported	Significantly increased Bax/Bcl-2 ratio, suggesting increased susceptibility to apoptosis; mechanism involves AMPK activation and inhibitor of Akt/mTOR signaling	Concentration-dependent reduction in cell survival (significant from 50 µM, maximum effect at 300 µM); decreased migration, reduced invasive capacity	[152]

**Table 11:** Summary of experimental studies investigating the effects of selected isoflavones and isoflavones-based combinations on p53 signaling, Bcl-2 family proteins, and apoptosis-related outcomes in cancer models.

Compound	Experimental Model (Cells/Organism)	Effect on p53	Effect on Bcl-2/Apoptotic Pathway	Biological Outcome (Cell Cycle/Apoptosis)	Source
Phenozodiol	Me4405 (p53-wild-type, Phenoxodiol-sensitive)	p53 protein markedly up-regulated after 12 h treatment	↑ BH3-only proteins Bad, PUMA, Noxa (p53-dependent) and Bim (p53-independent); Bax conformational change and activation; ↓ anti-apoptotic Bcl-xL and XIAP; Bcl-2 levels unchanged but over-expression blocks apoptosis	Strong apoptosis (12–48% at 48 h) with caspase-3/-9 activation, PARP and ICAD cleavage; modest p21 rise, no pronounced G_1_-S arrest	[153]
	Mel-RM (p53-wild-type, Phenoxodiol-resistant)	No significant change in p53 after treatment	↑ BH3-only proteins Bad, PUMA, Noxa not induced; Bim still up-regulated; limited Bax activation; Bcl-2 over-expression further suppresses apoptosis	Low apoptosis despite caspase activation; mitochondrial membrane potential only modestly altered	
	Mel-AT (p53-wild-type, Phenoxodiol-sensitive)	Similar p53 up-regulation as Me4405 (implied from “sensitive lines”)	↑ Bad, PUMA, Noxa, and Bim; Bax activation; ↓ Bcl-xL and XIAP	Apoptosis comparable to Me4405; caspase-3/-9 dependent	
Warangalone	HeLa cervical cancer cells (in vitro)	Warangalone increases p53 phosphorylation/activation	Down-regulates anti-apoptotic Bcl-2 and Bcl-XL while up-regulating pro-apoptotic Bax and Bad; this disrupts mitochondrial membrane potential, releases cytochrome C, and activates caspase-9 and caspase-3	Induces mitochondria-mediated (intrinsic) apoptosis, as evidenced by increased Annexin V-positive cells, PARP cleavage, and loss of cell viability	[154]
Genistein	Human localized prostate cancer patients (laser-capture-microdissected malignant and benign luminal cells)	Slight down-regulation of p53 mRNA; protein levels unchanged and not statistically significant	No significant change in Bcl-2 protein expression (genistein vs. placebo)	No significant effect on apoptosis-related markers (BAX, Bcl-2) or proliferation (Ki-67); overall biomarkers of cell-cycle progression unchanged	[155]
Genistein	SW480 (primary colorectal adenocarcinoma)	Not detected at 24 h or 48 h	No levels of caspase 3, cleaved PARP, cytochrome c, or Bcl-2 were detected; high ROS production was observed at 24 h	77.8% cell death at 24 h, 21.9% at 48 h; hypothesized necroptosis via ROS rather than caspase-mediated apoptosis	[156]
	SW620 (metastatic colorectal adenocarcinoma)	Significant increase in p53 at 48 h, not at 24 h	Bcl-2 unchanged at 48 h (not detected at 24 h); significant increases in cytochrome c, caspase 3, and cleaved PARP at both time points	44.2% cell death at 24 h, 30.3% at 48 h; intrinsic apoptosis involving mitochondrial membrane permeabilization, caspase activation, and ROS	
Genistein	HeLa human cervical cancer (HPV 18 positive)	P53 signaling pathway identified as one of the enriched KEGG pathways from network pharmacology prediction	Apoptosis pathway and “regulation of programmed cell death” identified as enriched by KEGG/GO analysis; genistein is known to involve both extrinsic and intrinsic apoptotic pathways and mitochondrial apoptosis in cervical cancer cells, though Bcl-2/Bax were not directly measured here	Strongly inhibited cell viability and proliferation (12.5–100 µM, 24–48 h); reduced colony formation; inhibited cell adhesion, migration and invasion; prior studies report G_2_/M phase arrest	[157]
Isoflavones from Soybean Cake	LNCaP prostate cancer cells	p53 protein expression was increased after treatment with the aglycon fraction, genistein, and the genistein + daidzein combination	Bcl-2 expression was not significantly changed by any isoflavone treatment; the rise in sub-G_0_/G_1_ population indicates apoptosis	Cell-cycle arrest at G_2_/M (high G_2_/M ratio) and increased sub-G_0_/G_1_ (apoptotic) fractions after isoflavone exposure	[158]
	PC-3 prostate cancer cells	No detectable change in p53 expression after isoflavone treatment	Bcl-2 expression was largely unchanged; a slight decrease was observed only with the aglycon fraction	Predominant G_2_/M arrest at higher isoflavone concentrations and a marked rise in sub-G_0_/G_1_ cells, especially with the aglycon fraction, indicating apoptosis	
Isoflavones from Chickpea *Cicer arietinum* L.	SKBr3 (ER-negative)	Dose-dependent increase in P53 protein expression (≈2.8-fold vs. control)	↓ Bcl-2 mRNA; ↑ Bax mRNA; ↓ Bcl-2/Bax ratio; ↑ caspase-7 and -9 protein; ↑ P21 (cell-cycle inhibitor)	Mitochondria-dependent apoptosis (↑ Annexin V/PI, morphological changes) and cell-cycle arrest via P21 up-regulation	[159]
	MCF-7 (ER-positive)	Dose-dependent increase in P53 protein (≈6.8-fold vs. control)	↓ Bcl-2 mRNA; ↑ Bax mRNA; ↓ Bcl-2/Bax ratio; ↑ caspase-7 and -9 protein; ↑ P21 (≈3.6-fold)	Same mitochondrial apoptosis and P21-mediated cell-cycle inhibition as SKBr3	
Jaceosidin	Human NSCLC A549 cells (in vitro)	Not directly assessed in this study	Bax ↑, Bcl-2 ↓ (dose- and time-dependent) Cytochrome c released to cytosol ↑ Cleaved caspase-9 ↑ (intrinsic pathway) Cleaved caspase-8 ↑ (extrinsic pathway) Cleaved caspase-3 ↑, Cleaved PARP ↑ → Both intrinsic mitochondrial and extrinsic death receptor pathways activated	S-phase cell cycle arrest (p21 ↑) Cell viability ↓ (MTS assay; IC50 = 55 µM in A549 vs. 248.5 µM in 293T—4.52-fold tumor-selective) Apoptosis rate ↑ (early + late), marked at 120 µM Ras/Raf/MEK/ERK and Akt pathways ↓	[160]

**Table 12:** Comparative overview of flavonoid subclasses and their modulation of the p53–Bcl-2 apoptotic axis.

Flavonoid Subclass	Representative Compounds	p53 Modulation	Bcl-2 Family Modulation	ROS Involvement	Main Apoptotic Outcome
Flavonols	Quercetin, Kaempferol, Myricetin	Strong activation/stabilization of p53	↑ Bax, ↓ Bcl-2, ↓ Bcl-xL	Moderate to high	Mitochondrial apoptosis, caspase activation
Flavones	Apigenin, Luteolin, Chrysin	p53 activation in several models	↑ Bax/Bcl-2 ratio	Moderate	Cell-cycle arrest and apoptosis
Flavanones	Naringenin, Hesperetin	Variable, partly p53-dependent	↑ Bax, ↓ Bcl-2	Moderate	Mitochondrial dysfunction and apoptosis
Anthocyanins	Cyanidin, Delphinidin	Limited evidence	Modulation reported in selected models	High	ROS-mediated apoptosis
Flavanols	EGCG, Epicatechin	p53 activation and stabilization	↑ Bax, ↓ Bcl-2	High	Caspase-dependent apoptosis
Isoflavones	Genistein, Daidzein	p53 activation and cell-cycle regulation	↑ Bax/Bcl-2 ratio	Moderate	Apoptosis and growth inhibition

## Data Availability

No new data were created or analyzed in this study. Data sharing is not applicable to this article.
